# PRRX1‐induced epithelial‐to‐mesenchymal transition in salivary adenoid cystic carcinoma activates the metabolic reprogramming of free fatty acids to promote invasion and metastasis

**DOI:** 10.1111/cpr.12705

**Published:** 2019-10-27

**Authors:** Ya‐ping Jiang, Ya‐ling Tang, Sha‐sha Wang, Jia‐shun Wu, Mei Zhang, Xin Pang, Jing‐biao Wu, Yu Chen, Ya‐Jie Tang, Xin‐hua Liang

**Affiliations:** ^1^ State Key Laboratory of Oral Diseases & National Clinical Research Center for Oral Diseases Department of Oral and Maxillofacial Surgery West China Hospital of Stomatology (Sichuan University) Chengdu China; ^2^ Department of Implant The Affiliated Hospital of Qingdao University Qingdao China; ^3^ State Key Laboratory of Microbial Technology Shandong University Qingdao China; ^4^ Key Laboratory of Fermentation Engineering (Ministry of Education) Hubei Provincial Cooperative Innovation Center of Industrial Fermentation Hubei Key Laboratory of Industrial Microbiology Hubei University of Technology Wuhan China

**Keywords:** free fatty acids (FFAs), metabolism, metastasis, PRRX1, salivary adenoid cystic carcinoma (SACC)

## Abstract

**Objectives:**

Increasing evidences demonstrate a close correlation between epithelial‐to‐mesenchymal transition (EMT) induction and cancer lipid metabolism. However, the molecular mechanisms have not been clarified.

**Materials and methods:**

In our study, the relative expression level of PRRX1 was detected, its relationship with free fatty acid (FFA) and PPARG2 was analysed in 85 SACC tissues and 15 salivary glands from the benign salivary tumours. We also compared the FFAs composition and levels in these SACC cells. PPARG2 was detected in PRRX1‐induced FFAs treatment as well as Src and MMP‐9 were detected in FFAs treatment–induced invasion and migration of SACC cells, and ChIP test was performed to identify the target interactions.

**Results:**

Our data showed that overexpression of PRRX1 induced EMT and facilitated the invasion and migration of SACC cells, and PRRX1 expression was closely associated with high FFAs level and poor prognosis of SACC patients. Furthermore, PRRX1 silence led to the increase of PPARG2 and the reduction of FFAs level and the migration and invasion of SACC cells. And inhibition of PPARG2 rescued FFAs level and migration and invasion capabilities of SACC cells. Free fatty acids treatment induced an increase of Stat5‐DNA binding activity via Src‐ and MMP‐9‐dependent pathway**.**

**Conclusions:**

Collectively, our findings showed that the PRRX1/PPARG2/FFAs signalling in SACC was important for accelerating tumour metastasis through the induction of EMT and the metabolic reprogramming of FFAs.

## 1 INTRODUCTION

Salivary adenoid cystic carcinoma (SACC) is one of the most common highly malignant salivary gland tumours and accounts for 10% of salivary gland tumours and 21%‐24% of adenocarcinoma and overall <1% of all cancers in the United States or <3% in China.^1^, ^2^, ^3^ Five‐year survival rate for patients with SACC is >70% but drops to 50% after 10 years, and 20% after 20 years when invasion into adjacent tissues and haematogenous spread to distant organs (lung, bone and liver). Approximately 40% of SACC patients have distant metastases, and metastasis is one of the most common poor prognostic factors in SACC patients.^4^

Epithelial‐mesenchymal transition (EMT) in cancer cells has been considered to be not a simple process to acquire invasive and metastatic abilities,^5^, ^6^, ^7^, ^8^ but a complicated and comprehensive reprogramming, involved in metabolism, epigenetics and differentiation.^9^ Metabolic reprogramming of cells has long been appreciated to contribute to oncogenesis.^10^, ^11^ Alteration in free fatty acids (FFAs) metabolism in cancer cells is one of the most important parts of the lipid metabolism.^12^ However, the molecular mechanism underlying lipid metabolic changes during EMT and metastatic progression of cancer, including alterations in regulatory gene expression, still remain undefined.

Free fatty acids, either saturated or unsaturated, consist of the terminal carboxyl group and the hydrocarbon chain mostly occurring in even numbers of carbons.^13^ Epidemiologic study exhibited that there was negative association between breast cancer and colon cancer patients and cis‐mono‐unsaturated fatty acids and positively with trans fatty acids.^14^ An altered free fatty acid pattern played a detrimental role in the progression of colorectal and prostate cancer patients.^15^, ^16^ Elongation of fatty acids from C16 to C18 had been reported to promote both hepatic lipid accumulation and inflammation.^17^ G‐protein‐coupled receptor 120 (GPR120) and GPR40 were identified as FFA receptors (FFARs) for long‐ and medium‐chain fatty acids. GPR120 negatively and GPR40 positively regulated cellular functions during the malignant progression of lung cancer.^18^ These indicated that changed FFAs could affect the biological behaviours and malignant characteristics of cancer cells. However, the epidemiological evidence of the correlation between increased FFAs and poor SACC prognosis has been unknown.

PRRX1, the paired‐related homeobox transcription factor, is an EMT inducer conferring the migratory and invasive properties of cancer cells.^19^, ^20^ In pancreatic ductal adenocarcinoma, PRRX1b promoted tumour invasion, dedifferentiation and EMT. And PRRX1a stimulated metastatic outgrowth in the liver, tumour differentiation and mesenchymal‐epithelial transition (MET).^21^ On the other side, the global gene expression assay in abdominal subcutaneous adipose tissue showed that homeobox transcription factors (such as PRRX1) and extracellular matrix structural proteins were upregulated after fat loss, implicating PRRX1 as an important regulator of adipose tissue function.^22^ Integrative computational analysis of phylogenetic conservation detected that PRRX1 contributed to the release of FFAs and insulin resistance.^23^ The data indicated that PRRX1 was an EMT inducer of cancer cells, and PRRX1 can mediate lipid metabolism of adipose tissue; however, to our knowledge, whether PRRX1 could regulate lipid metabolism to involve in the invasion and metastasis of cancer cells has yet not been reported. Importantly, in breast cancer cells undergoing EMT, an increased expression of fatty acid synthase (FASN) resulted in saturated fatty acids (FAs) accumulation and relocation through activating the EMT inducer VEGF/VEGFR2 signalling.^24^ The elevated free fatty acid uptake via CD36 was associated with EMT induction in hepatocellular carcinoma.^25^ These results suggest that EMT may change FFAs to regulate lipid metabolism of cancer cells during cancer progression. We therefore hypothesize that PRRX1 is important for tumour invasion and metastasis through both the induction of EMT and the metabolic reprogramming of FFAs.

## 2 MATERIALS AND METHODS

### 2.1 Cell culture

SACC‐83 and SACC‐LM cells (Figure S6) were obtained from the State Key Laboratory of Stomatology, Sichuan University, China. Both cell lines were cultured in high glucose DMEM (HyClone) supplemented with 10% foetal bovine serum (Thermo Fisher Scientific), 100 U/mL penicillin (Beyotime Biotechnology) and 100 U/mL streptomycin (Beyotime Biotechnology) at 37°C in a humidified atmosphere containing 5% CO_2_. Experiments were performed with cells undergoing logarithmic growth.

### 2.2 Patients and sample collection

Paraffin‐embedded sections of 85 SACC patients, who underwent resection of their tumours without preoperative chemotherapy, hormone therapy or radiotherapy, and 15 salivary glands from the benign salivary tumour patients were obtained from the Department of Oral and Maxillofacial Surgery, West China Hospital of Stomatology, Sichuan University, between 2000 and 2006. The principal clinicopathologic features of SACC patients are summarized in Table 1. In addition, the fresh tissues of 25 cases, diagnosed as SACC with PRRX1‐positive staining in immunohistochemistry, were obtained from the Department of Oral and Maxillofacial Surgery, West China Hospital of Stomatology, Sichuan University, between 2014 and 2016. The protocol of the study was approved by the Institutional Ethics Committee of the West China Medical Center, Sichuan University, China.

### 2.3 Immunohistochemistry

Fresh tissue samples were fixed in 10% formaldehyde and embedded in paraffin. Sections were cut and stained using a conventional immunohistochemistry procedure. Sections were incubated with PRRX1 antibody (1:80 dilutions; Novus Biologicals) or PPARG2 antibody (1:50 dilutions; ProteinTech) for 2 hours, followed by incubation with secondary antibodies (DAKO) for 30 minutes.

### 2.4 Lenti virus transfection and confirmation of transfection

The PRRX1 overexpressed lentiviral vector with a luciferase reporter gene was synthesized by Guangzhou Cyagen Biosciences Inc It was constructed by ligating the human PRRX1 sequence (654 bp) into the *Bam*HI/*Asc* II sites of the pLV.ExBi.P/Puro‐EF1α‐PRRX1‐IRES‐luc2 (8934 bp). The lentiviral vector was packaged using pCD/NL‐BH*DDD packaging plasmid mix (Addgene) and transiently cotransfected into 293T cells to generate recombinant virus particles. After 48 hours of infection, lentivirus in the supernatant was transduced into SACC cells, using 5 μg/mL of polybrene (Sigma‐Aldrich, Germany) at the optimal MOI (multiplicity of infection) of each cell. Stable clones were maintained on 5 μg/mL of puromycin (Sigma‐Aldrich). Fluorescence intensity of D‐luciferin was observed by fluorescence microscope to indicate the lentivirus transfection efficiency.

### 2.5 Real‐time RT‐PCR

Total RNA was extracted from cells using the Trizol (Invitrogen). The PCR conditions used were initial denaturation at 95°C for 15 minutes, followed by 45 cycles of denaturation at 94°C for 15 seconds, annealing at 60°C for 25 seconds and extension at 72°C for 15 seconds. The calculation formula was ΔΔCt = (Ct^A2^ − Ct^B2^) − (Ct^A1^ − Ct^B1^). The primer sequences for PRRX1, forward primer: 5′‐TATCTCTCCTGGGGGACAGC‐3′, reverse primer: 5′‐CGTTATGAAGCC CCTCGTGT‐3′, for PPARG2 forward primer: 5′‐AGCCCTTCACTACTGTTG ACTTCTC‐3′ and reverse primer: 5′‐CTTTGATTGCACTTTGGTACTCTTG‐3′, and GAPDH forward primer: 5′‐ATGGGGAAGGTGAAGGTCG‐3′ and reverse primer 5′‐TAAAAGCAGCCCTGGTGSACC‐3′.

### 2.6 Western blot

Thirty microgram of proteins from each sample was separated on 8% sodium dodecyl sulphate‐polyacrylamide gel electrophoresis (SDS‐PAGE) and transferred electrophoretically to polyvinylidene difluoride (PVDF) membranes (Millipore). Membranes were blocked with 4% bovine serum albumin (BSA) and then incubated for 2 hours with rabbit anti‐PRRX1 (1:1000 dilutions; Abcam), rabbit anti‐PPARG2 antibody (1:500 dilutions; ProteinTech), rabbit anti‐E‐cadherin (1:500 dilutions; Abcam), rabbit anti‐vimentin (1:500 dilutions; Abcam), rabbit anti‐Snail1 (1:1000 dilutions; Abcam), mouse anti‐GAPDH (1:1000 dilutions; ProteinTech), rabbit anti‐Beta‐actin (1:500 dilutions; ProteinTech). The procedure was done following manufacturer's instructions.

### 2.7 Immunofluorescence

Cells were cultured at a density of 3 × 10^4^ cells per chamber. Upon reaching 70% confluency, culture media was removed, washed and fixed. Cells were incubated with PRRX1 antibody (1:250 dilutions; Abcam), PPARG2 antibody (1:100 dilutions; ProteinTech) and Alexa Fluor^®^ 488‐conjugated secondary donkey anti‐rabbit antibody (1:1000 dilutions; Abcam). Cells were visualized using the Olympus FluoView™ confocal microscope (Tokyo, Japan), and confocal fluorescence images were taken. Cells were recorded in six different microscopic fields.

### 2.8 Gas chromatography‐mass spectrometry analysis (GC/MS)

All analyses were performed in split mode (1:20) on an Agilent 7890a gas chromatograph connected to an Agilent 5975C Series MSD (Agilent Technologies). The chromatographic columns were 30 m DB‐5 MS + DG capillary columns (5% phenyl, 95% dimethylpolysiloxane) with an internal diameter of 250 μm (Agilent Technologies) and a 25 m × 0.25 mm SLB‐IL82 column with a film thickness of 0.2 μm (Supelco). The injection volume was 1 μL. The MS source and MS quadrupole were maintained at 230°C and 150°C, respectively. The masses of the analytes were acquired in full‐scan mode with mass range of 30‐650 m/z.

### 2.9 Free fatty acid quantification colorimetric/fluorometric kit

Free fatty acid quantification was performed using a free fatty acid quantification colorimetric/fluorometric kit (BioVision).^26^ The palmitic acid standard liquids were diluted to 0, 0.2, 0.4, 0.6, 0.8, 1.0 nmol/well. 1 × 10^6^ SACC‐LM or SACC‐83 cells or 10 mg SACC tissue samples were extracted by homogenization with 200 µL of chloroform‐Triton X‐100. The extracts were spined 10 minutes and dried to remove trace chloroform. The dried lipids were dissolved in 200 µL of fatty acid assay buffer. The enough reagents were mixed for the number of assays and standard performed according to the manufacturer's instructions. OD 570 nm was used for colorimetric assay or Ex/Em = 535/590 nm was used for fluorescence in a microplate reader.

In SACC tissues, the FFA concentration of the normal salivary gland tissues was designated as the relative baseline. Compared with the baseline, the FFAs concentration was divided into the low FFAs group and the high FFAs group.

### 2.10 Wound healing assay

Cells cultured in serum‐free media for 24 hours in wound healing assays. Cells were plated in 6‐well plates at 2.0 × 10^5^ cells/ well. The individual wells were wounded by scratching with a pipette tip and incubated with medium containing no FBS to 24 hours. Wound closures were photographed to measure the remaining cell‐free area in triplicates wells. The percentage of the cell‐free area was calculated and showed as mean ± SD of three independent experiments.

### 2.11 Transwell invasion assay

The invasion assay was conducted using 1.5 × 10^5^ cells and Matrigel‐coated membrane (24‐well insert, pore size, 8 μm; BD Biosciences). After incubated for 24 hours, migrated cells on the lower surface of the membrane were stained with 0.1% crystal violet. Five fields per filter were counted.

### 2.12 Chromatin immunoprecipitation (ChIP) assays

ChIP assays were performed using a ChIP Assay Kit (Abcam) according to the manufacturer's instructions. Briefly, the minimum number of SACC‐LM or SACC‐83 cells was 1 × 10^6^ cells for every ChIP. These cells were fixed and lysed, and the extracted DNA was sheared by the sonicator to an optimal DNA fragment size of 200‐1000 bp. Chromatin was then precipitated with nonspecific IgG antibodies (Sigma), ChIP‐grade PRRX1 antibodies (Proteintech) or ChIP‐grade rabbit anti‐histone H3 (Sigma). Then, DNA was extracted and purified, and PCR was performed with primers for a PPARG2 promoter fragment.

### 2.13 Xenograft tumour model

The mice were randomly distributed into four groups (n = 5): injected subcutaneously + SACC‐83 vector, injected subcutaneously + SACC‐83 PRRX1, injected intravenously + SACC‐83 vector, injected intravenously + SACC‐83 PRRX1. After a week's acclimation, luciferase‐labelled cells (1.0 × 10^6^) were suspended in 100 μ L of Hank's buffered salt solution and injected subcutaneously and intravenously via the tail vein. The mice were monitored daily for body weight, behaviour and water/food consumption. Tumour growth was monitored by measuring the tumour size in two orthogonal dimensions with vernier calipers every 4 days and the tumour volume was calculated.

The incidence and volume of metastases were estimated by serial imaging of mice for bioluminescence using the IVIS 100 imaging system coupled to a data‐acquisition personal computer equipped with Living Image software (Xenogen). The photon emission level (representative of luciferase activity) was used to assess the relative tumour burden in the mice. After tumour cells injection for 4 weeks, the mice were anaesthetized and sacrificed. The tumours were surgically excised, weighed, fixed in 10% formalin and embedded in paraffin.

### 2.14 Statistical analysis

All statistical analyses were performed using the SPSS package (version 17.0). A value of *P* < .05 was considered statistically significant.

## 3 RESULTS

### 3.1 PRRX1 is positively associated with FFA accumulation in salivary adenoid cystic carcinoma patients

**Figure 1:**
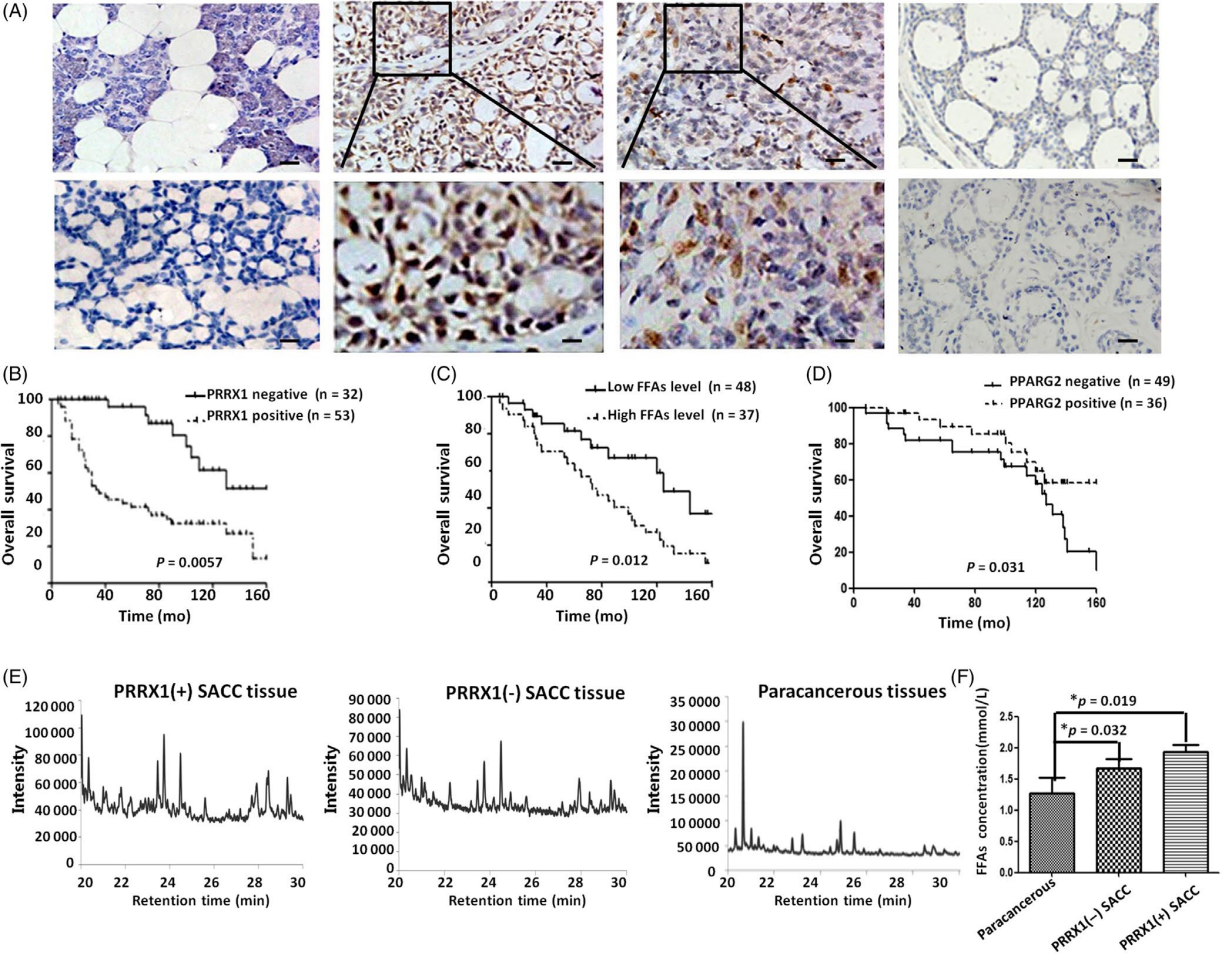
PRRX1 was significantly associated with FFA accumulation in SACC patients. A, The expression of PRRX1 in SACC and the normal parotid tissue with IHC. First column: PRRX1‐negative staining of normal salivary gland tissue (top ×200) and PRRX1‐negative staining of SACC (bottom ×200); second column: PRRX1‐positive staining of tube pattern of SACC (top ×200) and the magnification of PRRX1‐positive staining of tube pattern of SACC (bottom ×400); third column: PRRX1‐positive staining of solid pattern of SACC (top ×200) and the magnification of PRRX1‐positive staining of solid pattern of SACC (bottom ×400); fourth column: PPARG2‐positive staining in PRRX1‐negative SACC samples (top ×200) and PPARG2‐negative staining in PRRX1‐positive SACC samples (bottom ×200). The number of SACC samples was 85, and the number of salivary gland samples from the benign salivary tumours was 15. B, The overall survival curves in PRRX1 negative (n = 32) or positive (n = 53) of SACC (*P* = .0057). C, The overall survival curves in SACC tissue with low (n = 48) or high level (n = 37) of FFAs (*P* = .012). D, The overall survival curves in PPARG2 negative (n = 49) or positive (n = 36) of SACC (*P* = .031). E, The GC/MS chromatograms of composition of FFAs among PRRX1 positive SACC (n = 53), PRRX1‐negative SACC (n = 32) and paracancerous tissue (n = 15), respectively. There was statistically different FFAs composition a PRRX1‐positive SACC, PRRX1‐negative SACC and paracancerous tissue (*P < .05). Compared with paracancerous tissues and SACC with negative PRRX1, the composition of FFAs of PRRX1‐positive SACC was more complex, and the carbon chain from C16 to C18 extend product increased (*P < .05). F, The levels of FFAs in PRRX1‐positive SACC, PRRX1‐negative SACC, and paracancerous tissue, respectively. The FFAs quantitative analysis displayed that the FFAs levels of SACC tumour tissues with PRRX1 negative were significantly higher than tissues adjacent to the benign salivary tumours (*P* = .032), and the FFAs level of PRRX1‐positive group was significantly higher than PRRX1‐negative group (*P* = .019). Error bars represent the mean ± SD of triplicate experiments. One‐way ANOVA statistical analysis was performed to determine significance

To evaluate the clinical outcomes of PRRX1 in SACC, we examined the expression of PRRX1 in 85 salivary adenoid cystic carcinoma patients who received surgery in the West China Hospital of Stomatology, Sichuan University, in China by immunohistochemical staining. The PRRX1‐positive staining was mainly located in nucleuses of cancer cells (Figure 1A). The positive rate of PRRX1 in SACC cases was 62.35% (53/85), while only 20% (1/5) in normal salivary tissues. The positive expression of PRRX1 was significantly associated with location, pathological classification, TNM stage, local invasion, and distant metastasis and recurrence of SACC patients (*P* < .05, Table 1). Fifty‐three patients with PRRX1‐positive expression had a significantly worse prognosis than PRRX1‐negative patients (*P* < .05, Figure 1B). And in Oncomine database, the expression of PRRX1 was also upregulated in 16 cases of salivary adenoid cystic carcinoma and 15 cases of oral squamous cell carcinoma (Figure S1A‐C). This indicated that PRRX1 may be one of the important markers in SACC patients.

**Table 1:** Clinical‐pathologic characteristic of 85 patients with SACC and association between PRRX1 expression and these variables

Clinical‐pathologic variables	No. of patients	PRRX1 expression		*P* value
		Negative (%)	Positive (%)	
Age				
<50	34	13(38.24)	21(61.76)	.819
≥50	51	19(37.25)	32(62.75)	
Gender				
Male	47	17(36.17)	30(63.83)	.824
Female	38	15(39.47)	23(60.53)	
Location				
Major	40	21(52.50)	19(47.50)	**.013**
Minor	45	11(24.44)	34(75.56)	
Histological subtype				
Pore/tube	62	28(45.16)	34(54.84)	**.024**
Solid	23	4(17.39)	19(82.61)	
TNM stage				
I‐II	26	15(57.69)	11(42.31)	**.015**
III‐IV	59	17(28.81)	42(71.19)	
Perineural invasion				
Positive	38	18(47.37)	20(52.63)	**.014**
Negative	47	35(74.47)	12(25.53)	
Metastasis				
Positive	33	14(42.42)	19(57.58)	**.003**
Negative	52	39(75.00)	13(25.00)	
Recurrence				
Positive	23	10(43.48)	13(56.52)	**.043**
Negative	62	43(69.35)	19(30.65)	

The bold of *P* value means sigificant difference.

Recently, FFA metabolic reprogramming, which alters paradigm in energy metabolism as well as serves as mediators in signal transduction, plays an important role in cancer progression.^16^, ^27^ Here, we applied GC/MS to examine FFAs distribution in 85 SACC patients and analysed their association with the prognosis of patients to uncover the role of FFAs in SACC. The data showed that there was a significant correlation of tumour FFA level with both disease‐free survival (*P* = .022) and overall survival (*P* = .012; Figure 1C, Figure S1D). Compared with the normal salivary tissue and SACC cases with negative PRRX1, the composition of FFAs of SACC with positive PRRX1 was more complex, and the number of the carbon chain from C16 to C18 extended products added (*P* < .01, Figure 1E, Table S1). Moreover, the FFA quantitative analysis displayed that the FFA level in PRRX1‐positive group was significantly higher than in PRRX1 negative, and the FFA level in SACC cases was obviously higher than normal salivary tissue (*P* < .05, Figure 1F). This suggested that there may be a close relationship between PRRX1 and FFAs distribution in SACC patients. In addition, PPARG2 could control lipid metabolism of adipocytes including adipose cell differentiation, lipid storage, FFA transport and β‐oxidation.^28^ Therefore, we detected PPARG2 expression in SACC samples by immunohistochemical staining. The results showed that PPARG2 expression was negatively the expression of PRRX1 in SACC tissue (Figure 1A), and PPARG2 overexpression implied the good prognosis of SACC patients (*P* = .031; Figure 1D).

### 3.2 PRRX1 induces EMT to promote migration and invasion of salivary adenoid cystic carcinoma cells

**Figure 2:**
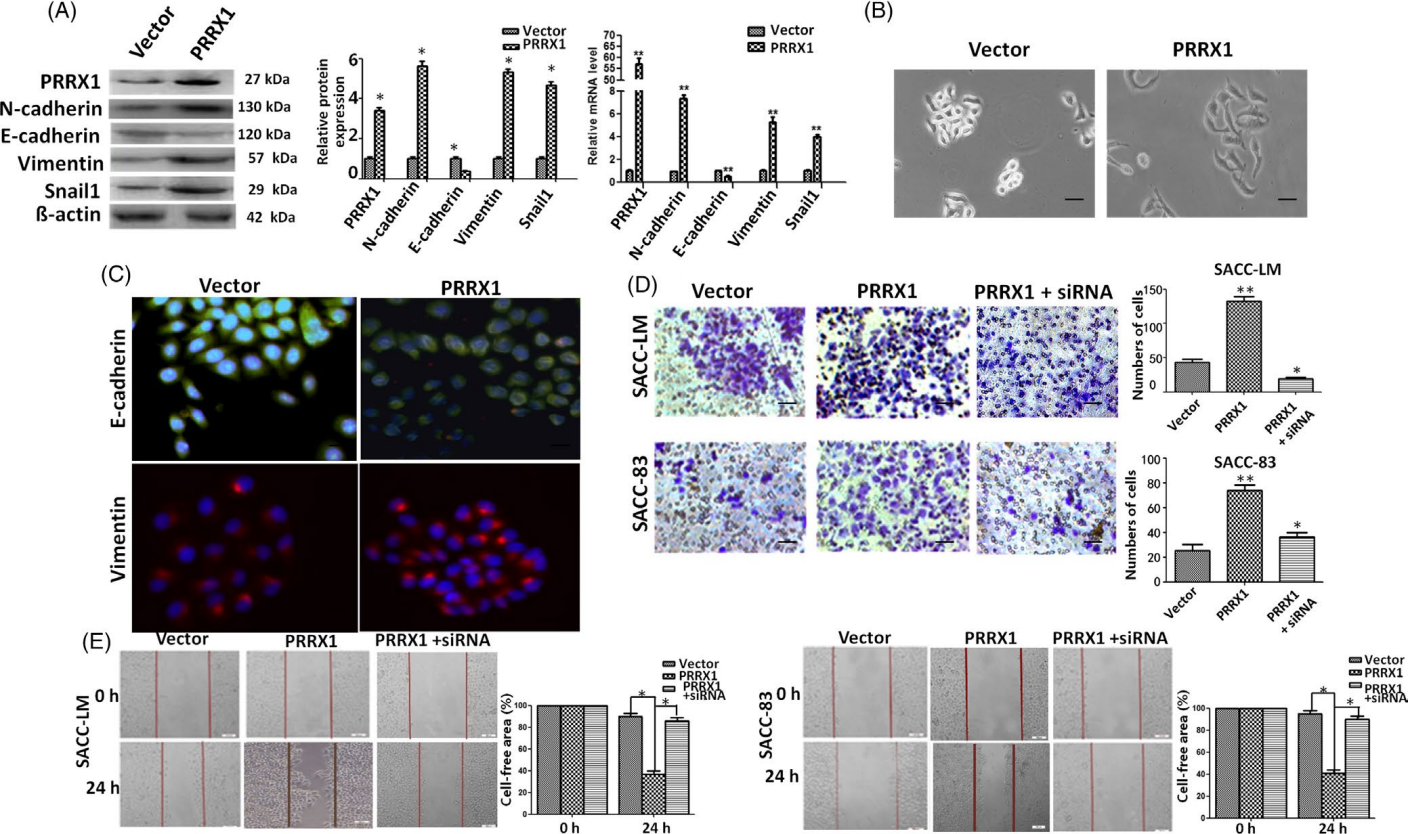
PRRX1 induced EMT to fuel migration and invasion of SACC cells. A, Immunoblotting and RT‐PCR analysis of the expression of E‐cadherin, N‐cadherin, vimentin and EMT transcription factor Snail1 in PRRX1 overexpressed SACC‐83 cells. The data showed that PRRX1 overexpressed SACC‐83 cells exhibited a significant downregulation of E‐cadherin and upregulation of N‐cadherin, vimentin and Snail1. Error bars represent the mean ± SD of triplicate experiments (*P < .05, **P < .001 compared with the vector). B, Morphologic change of SACC‐83 cells overexpressing PRRX1 or empty vector. Scale bar, 100 mm. C, Immunofluorescence staining for E‐cadherin and Vimentin in PRRX1 overexpressed SACC‐83 cells. Scale bar, 100 mm. D, E, Invasion D, and migration E, assays in stable PRRX1 lentiviral vector transfected SACC‐LM and SACC‐83 cells with or without PRRX1 siRNA. The data showed that the knockdown of PRRX1 reduced the migration and invasion abilities of PRRX1 overexpressed SACC cells, similar to those of the control. Each experiment was repeated 3 times. Representative images of migrated and invaded cells are shown. Error bars represent the mean ± SD of triplicate experiments. One‐way ANOVA statistical analysis was performed to determine significance

PRRX1 has been shown to be an EMT inducer in some kinds of cancers, but the role of PRRX1 in SACC remains unknown.^19^, ^20^ To examine the potential of PRRX1 to induce EMT in salivary adenoid cystic carcinoma, we applied the overexpressed lentiviral vector to transfect PRRX1 to salivary adenoid cystic carcinoma cells, SACC‐83 and SACC‐LM, which was confirmed by immunoblotting, real‐time PCR (Figure S2C). After cultured for 48 hours, the morphology of SACC‐83 and SACC‐LM cells became obviously stretched and elongated (Figure 2B). The epithelial biomarker E‐cadherin was downregulated, while simultaneously the mesenchymal biomarker N‐cadherin, Vimentin and EMT transcription factor Snail1 upregulated (Figure 2A) . Immunofluorescence staining showed weaker E‐cadherin membrane localization and stronger intracytoplasmic localization of Vimentin (Figure 2C). Furthermore, overexpression of PRRX1 in SACC‐83 and SACC‐LM cells significantly increased the invasive and migratory abilities (Figure 2D,E). Similarly, we used PRRX1 siRNA to transfect these SACC cells and found that knockdown of PRRX1 reduced the invasive and migratory abilities of SACC‐83 and SACC‐LM cells (Figure S2). These data indicated that PRRX1 might induce EMT in salivary adenoid cystic carcinoma cells.

Subsequently, we used PRRX1 siRNA to transfect SACC cells with overexpressed PRRX1 to knock down PRRX1 expression (Figure S2G,H). The silencing of PRRX1 in SACC cells rescued the expression of E‐cadherin and lowered the mRNA and protein levels of Vimentin and Snail1, almost up to the levels of the control. These changes were accompanied with a reduction of the migration and invasion capabilities of SACC cells, similar to those of the control (Figure 2D,E). The data further confirmed that PRRX1 might be an EMT inducer of salivary adenoid cystic carcinoma cells.

### 3.3 PRRX1 induces the accumulation of FFAs by mesenchymal cancer cells

**Figure 3:**
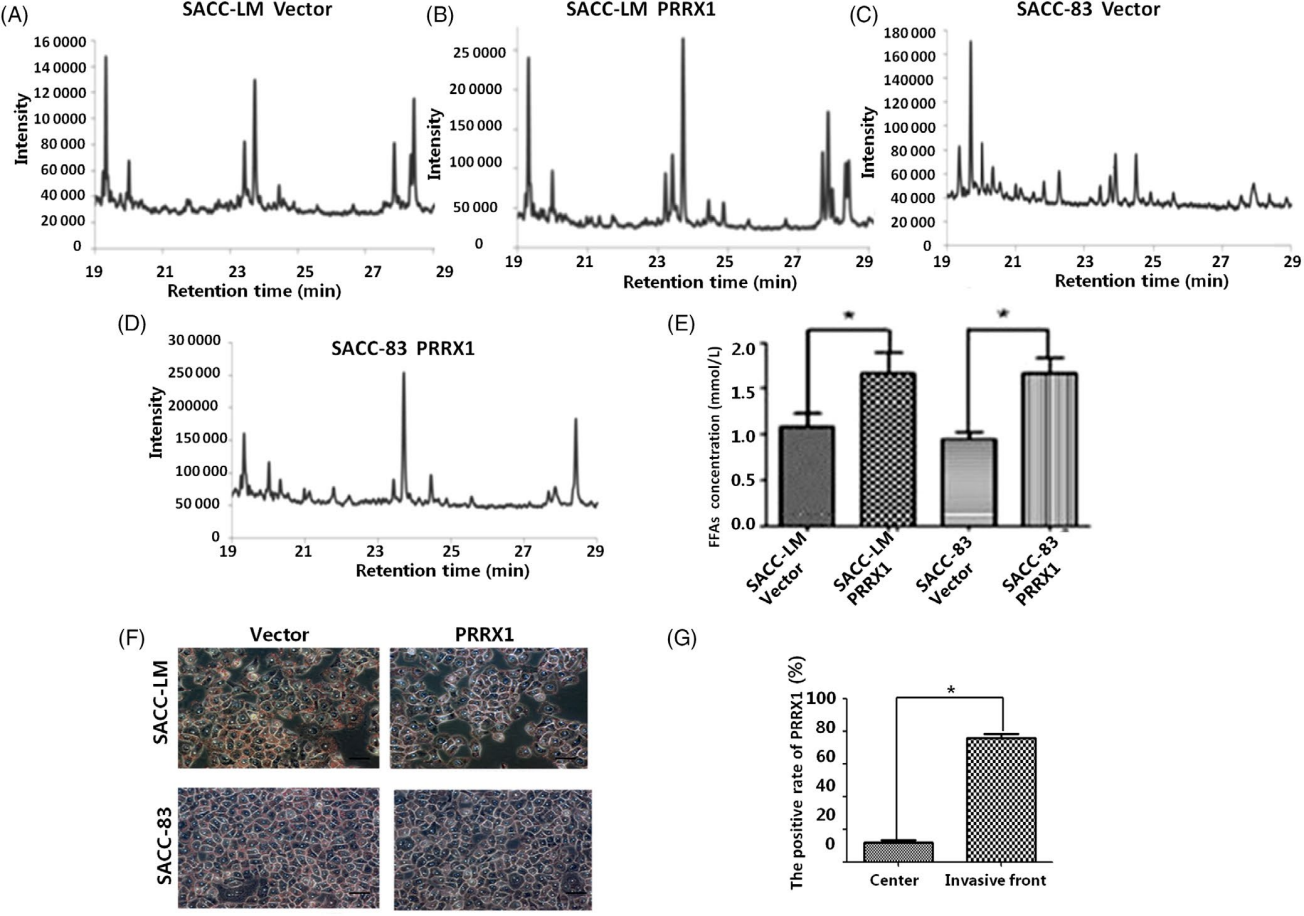
PRRX1 increased FFA level by mesenchymal cancer cells. A, B, The GC/MS chromatograms of free fatty acids in PRRX1 overexpressed SACC‐LM. In SACC‐LM cells, the composition of FFAs in PRRX1 overexpressed group was more complex than the vector, containing six different kinds of FFAs. C, D, The GC/MS chromatograms of free fatty acids in PRRX1 overexpressed SACC‐83 cells. In SACC‐83 cells, the composition of FFAs in PRRX1 overexpressed group was also more complex than the vector, containing five different kinds of FFAs, and carbon chain extension, the proportion of carbon 14 FFAs from 82.56% to 0.79%, the proportion of carbon 16 and 18 FFAs increased from 17.44% to 99.21%. E, The FFA quantitative comparison in PRRX1 overexpressed SACC‐LM and SACC‐83 cells with FFAs quantification colorimetric/fluorometric kit. The level of FFAs in PRRX1 overexpressed group was 1.54 times than the vector in SACC‐LM cells and 1.77 times in SACC‐83 cells (*P < .05). F, TG expression in PRRX1 overexpressed SACC‐LM and SACC‐83 cells of (oil red O staining ×200). G, PRRX1 expression in the centre of SACC and the invasive front of SACC invasion to gland with IHC. The data showed that the positive rate of PRRX1 at the invasive front was significantly higher than that at the centre of SACC (*P < .05). Error bars represent the mean ± SD of triplicate experiments. Unpaired *t* test was performed to determine significance

We next ascertained the relation between PRRX1‐induced EMT and FFAs in SACC cells. There were different proportions of saturated FFAs and unsaturated FFAs contained, and carbon chain length ranged from carbon 14 to 18 in SACC cells. In SACC‐LM cells, the composition of FFAs in PRRX1 overexpressed group was more complex than the vector, containing six different kinds of FFAs (Figure 3A,B; Table S2). In SACC‐83 cells, the composition of PRRX1 overexpressed group was also more complex, containing five different kinds of FFAs, and carbon chain extended. The proportion of carbon 14 FFAs reduced from 82.56% to 0.79%; the proportion of carbon 16 and 18 FFAs increased from 17.44% to 99.21% (Figure 3C,D; Table S2,). In addition, these changes were accompanied with an increase of the levels of FFAs (1.54‐fold SACC‐LM cells and 1.77‐fold in SACC‐83 cells) relative to that of the vectors (Figure 3E). In addition, it was reported an inverse relationship between FFAs and adipocyte triglyceride (TG) accumulation was observed in PRRX1‐overexpressed adipocytes.^24^ Hence, we detected the expression of TG in SACC‐LM and SACC‐83 cells with oil red O staining and both the proportion of stained cells and staining intensity were alleviated in PRRX1‐overexpressed group (Figure 3F). This also confirmed that PRRX1 may induce the expression of FFAs in SACC cells.

Then, we also compared the FFAs composition and levels in PRRX1 silenced SACC cells. In SACC‐LM cells, there were some saturated and unsaturated FFAs, while the proportion of unsaturated FFAs reduced from 23.63% to 7.41% in PRRX1 silenced group (Figure S3A; Table S3). In SACC‐83 cells, there were only saturated FFAs, while the proportion of carbon 14 FFAs increased from 73.63% to 91.38% in PRRX1 silenced group (Figure S3B; Table S3). Compared with the control, the concentration of FFAs decreased in PRRX1 silenced group and reduced to 53.50% in SACC‐LM and 52.99% SACC‐83 cells (Figure S3C, **P < .01）.The data indicated that there was a positive association of the concentration of FFAs and PRRX1 expression.

To evaluate the potential relationship of PRRX1 and FFAs in the tumour microenvironment, we analysed their expression in adjacent sections of SACC tissues by IHC staining for PRRX1 and GC/MS for FFAs. PRRX1 was expressed at higher levels at the invasive front (Figure 3G). We observed the same gradient for FFAs with higher levels at the tumour edge, where cancer cells displayed more mesenchymal features and expressed PRRX1 with a high level, compared with the centre of the tumour (Figure S3D,E). These data indicated that PRRX1‐induced mesenchymal cancer cells secreted more FFAs at the invasive front due to increased levels of PRRX1.

### 3.4 Downregulation of PPARG2 is required for upregulation of PRRX1 in response to FFAs treatment

**Figure 4:**
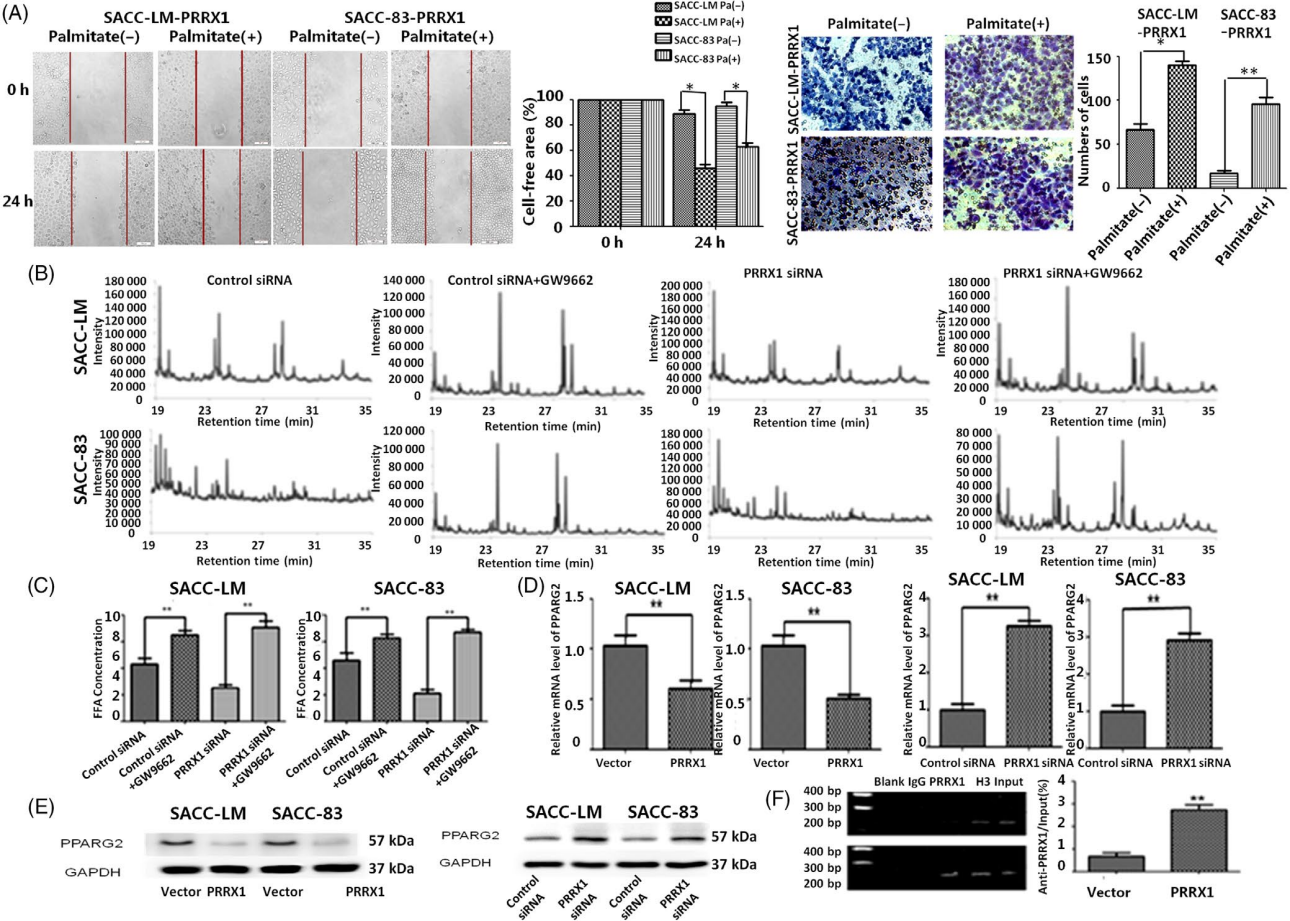
PRRX1 targeted regulating PPARG2 promoter and suppressed PPARG2 expression response to FFAs treatment. A, The PRRX1‐induced invasion and migration of SACC‐83 and SACC‐LM cells were enhanced in response to FFAs treatment. Pa: Palmitate. Error bars represent the mean ± SD of triplicate experiments. B, The GC/MS chromatograms of FFAs of the GW9662 stimulation SACC cells. Compared with the control group without GW9662, the composition of free fatty acids of the Rosiglitazone stimulating groups was more complex, and the long carbon chain extend product from C16 to C18 increased, and C19 and C20 emerged. C, The FFA level in the GW9662 stimulation of SACC cells by FFA quantification colorimetric/fluorometric kit (**P < .01). The FFA concentration of GW9662 stimulating group increased by 0.453 mmol/L and 1.107 mmol/L, respectively, in vector and PRRX1 groups in SACC‐LM cells, 0.411 mmol/L and 1.144 mmol/L, respectively, in vector and PRRX1 groups in SACC‐LM cells. D, The mRNA level of PPARG2 and PRRX1 was detected by RT‐PCR. The mRNA level of PPARG2 reduced to 52.17% and 49.63% in PRRX1 overexpressed SACC‐LM and SACC‐83 cells. The mRNA level of PPARG2 increased 3.17 and 2.95 times in PRRX1 siRNA groups of SACC‐LM and SACC‐83 cells, respectively. PPARG2 expression was significantly downregulated in PRRX1 overexpressed SACC‐LM and SACC‐83 cells, while PPARG2 expression was significantly upregulated by PRRX1 siRNA transfection (**P < .01). E, PPARG2 protein expression was detected by Western blot. The protein level of PRRX1 which aimed at PPARG2 was consistent with mRNA level. F, ChIP test showed that the combination capacity of PRRX1 and PPARG2 promoter in PRRX1 overexpressed group was significantly increased (**P < .01). Error bars represent the mean ± SD of triplicate experiments. Unpaired *t* test was performed to determine significance

Previous studies indicated that PPARG2 was involved in the regulation of FFAs metabolism of adipocytes and the progression of cancer cells.^29^, ^30^ PRRX1 has been shown to target and bind PPARG2 promoter in adipocytes.^24^ Hence, we first determined whether FFAs have an effect on PRRX1‐induced invasion and migration of SACC cells. The data showed that palmitate treatment augmented the PRRX1‐induced invasion and migration of SACC‐83 and SACC‐LM cells (Figure 4A). To further ascertain whether the effect of FFAs on the PRRX1‐induced invasion and migration was mainly dependent on PPARG2, we used Rosiglitazone, a specific PPARG2 agonist, to stimulate PPARG2 expression in PRRX1 overexpressed SACC cells, while the antagonist of PPARG2 (GW9662) to inhibit PPARG2 expression in PRRX1 silenced SACC cells.

In the Rosiglitazone group of SACC cells with PRRX1 overexpression, the constituent ratio of FFAs decreased and the percentage of long‐chain FFAs more than 16C reduced. And the FFAs concentration of Rosiglitazone group decreased (Tables S4 and S5, Figure S4A). In addition, Rosiglitazone significantly decreased the invasion and migration abilities of SACC cells with PRRX1 overexpression (Figure S4B). On the contrary, GW9662 in SACC cells with PRRX1‐silenced added the concentration and composition of FFAs, the long carbon chain extend product from C16 to C18 increased, and C19 and C20 emerged (Figure 4B,C; Tables S6 and S7). And GW9662 significantly promoted the invasion and migration of SACC cells with PRRX1‐silenced (Figure S5A,B). Collectively, these results suggest that PPARG2 plays a critical role in the regulation of PRRX1‐induced invasion and migration of salivary adenoid cystic cancer cells in response to FFAs.

Further, we examined whether there was the direct association between PRRX1 and PPARG2 expression. We found that PPARG2 dramatically reduced in PRRX1 overexpressed SACC cells, and PPARG2 increased in PRRX1‐silenced SACC cells (Figure 4D,E, Figure S5C). This indicated that PPARG2 expression was significantly inverse correlation with PRRX1. Moreover, the result of ChIP test confirmed that compared with the control group, PRRX1‐pparg2 compound of PRRX1 overexpressed group significantly increased in SACC‐83 and SACC‐LM cells (Figure 4F, **P < .01）, showing the combinative capacity of PRRX1 and PPARG2 promoter was significantly boosted. Taken together, these results suggest that PRRX1 may directly bind with PPARG2 promoter, inhibiting PPARG2 transcription in SACC cells.

### 3.5 FFAs treatment induces an increase of Stat5‐DNA binding activity via Src and MMP‐9‐dependent pathway

**Figure 5:**
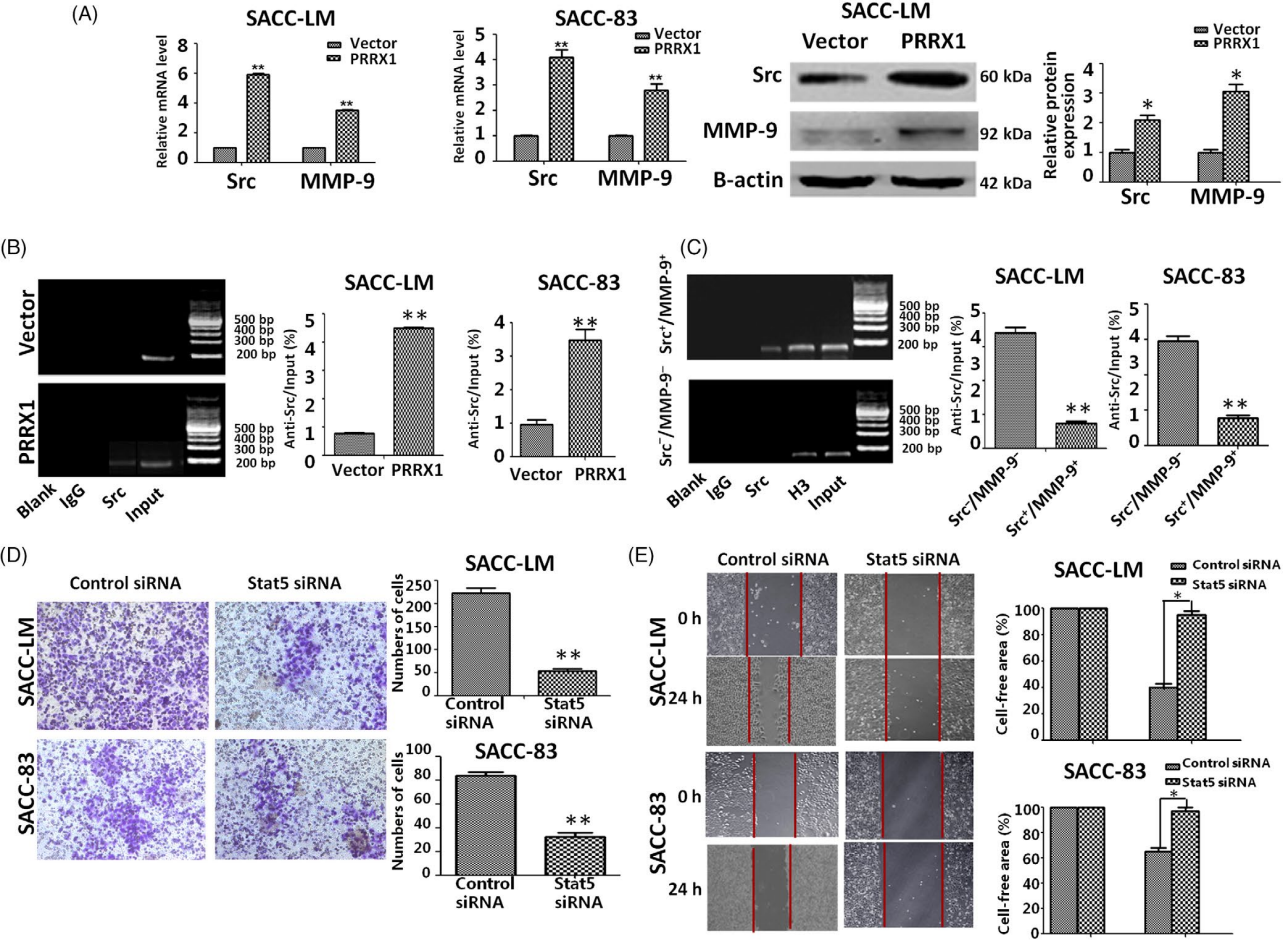
FFA contributes to the invasion and migration of SACC cells by inducing Stat5‐DNA binding activity via Src and MMP‐9‐dependent pathway. A, RT‐PCR and Western blot analysis of Src and MMP‐9 expression in PRRX1 overexpressed SACC‐83 cells. The data showed that Src and MMP‐9 expression significantly enhanced in PRRX1‐overexpressed SACC‐83 cells (**P < .01). B, The combination capacity of Src‐Stat5 compound significantly increased in PRRX1‐overexpressed SACC‐83 cells by ChIP test (**P < .01). C, When Src and MMP‐9 activities were inhibited, Stat5‐DNA binding activity was blocked (**P < .01). D, E, Invasion (D) and migration (E) assays in SACC‐LM and SACC‐83 cells with or without Stat 5 siRNA. The data showed that the knockdown of Stat5 reduced the migration and invasion abilities of SACC‐LM and SACC‐83 cells. Representative images of migrated and invaded cells are shown. The mean was derived from cell counts of 5 fields, and each experiment was repeated 3 times (*P < .05). Error bars represent the mean ± SD of triplicate experiments. Unpaired *t* test was performed to determine significance

It was reported that Src and MMP‐9 played important roles in arachidonic acid metabolism of MDA‐MB‐231 breast cancer cells.^31^ Here, we examined the role of Src and MMP‐9 activities in SACC cells with PRRX1‐induced FFAs accumulation. Accompanying FFAs accumulation in PRRX1 overexpressed SACC cells, we found that Src and MMP‐9 expression significantly were boosted (Figure 5A), and the precipitation of Src‐Stat5 compound significantly increased in SACC cells by ChIP test (Figure 5B). To ascertain that the effect of FFAs on the PRRX1‐induced invasion and migration was mainly dependent on Src and MMP‐9, SACC‐LM and SACC‐83 cells were treated for 30 minutes with 5 mM SU6656 and 10 mM GM6001, the selective inhibitors of Src and MMP‐9 activities, respectively, and then performed the stimulation with 100 mM palmitate for 30 minutes. As shown in Figure 5C, inhibition of Src and MMP‐9 activities completely dampened Src‐Stat5 binding activity induced by palmitate. Subsequently, we analysed the role of Stat5 on the migration and invasion of SACC cells. We used siRNAs against Stat5 to knock down the Stat5 expression in SACC cells. The migration and invasion of SACC cells were attenuated in Stat 5 siRNA groups by using Transwell and scratch‐wound assays (Figure 5D,E, *P < .05, **P < .01). This indicated that FFAs treatment might induce an increase of Src‐Stat5 binding activity via Src and MMP‐9‐dependent pathway to promote the invasion and migration of SACC cells.

### 3.6 PRRX1 and PPARG2 were responsible for lung metastasis of SACC‐83 cells in vivo

**Figure 6:**
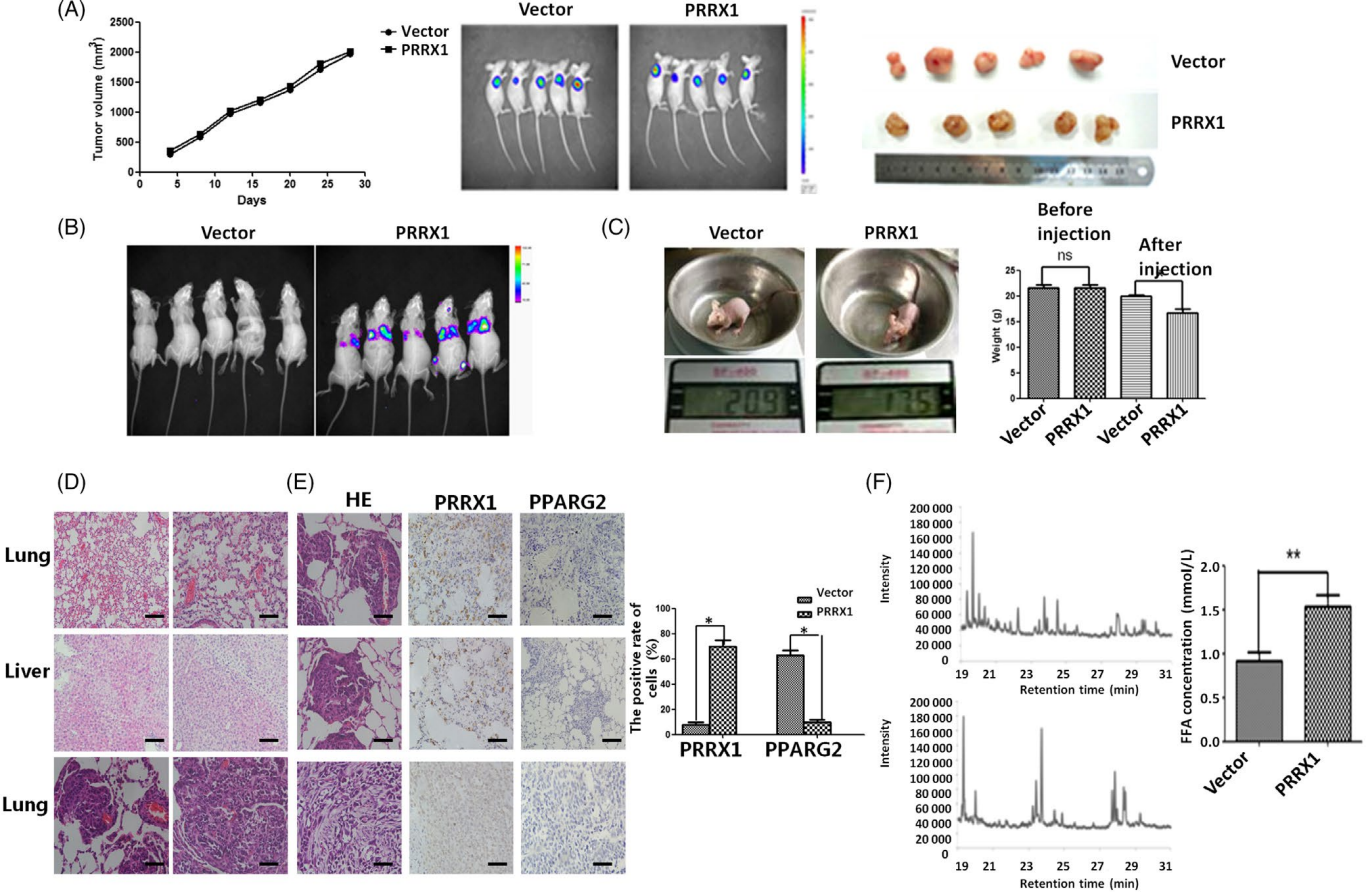
PRRX1 overexpression facilitated lung metastasis of SACC‐83 cells in vivo. A, The growth of nude mice‐bearing SACC after subcutaneously injecting SACC‐83 cells. Left column: The growth curve of nude mice‐bearing tumour volume in vector group and PRRX1 overexpressed group. The xenograft tumour volume was (2327 ± 227) mm^3^ in vector group and (2268 ± 203) mm^3^ in PRRX1 overexpressed group when 4 wk after subcutaneously injecting, and there was no significant difference in the tumour volumes between two groups (*P* = .91). Medium column: The average fluorescence area and intensity between vector group and PRRX1 overexpressed group had no significantly different through an in vivo imaging. Right column: The size of the gross specimen in vector group and PRRX1 overexpressed group of subcutaneously injecting SACC‐83 cells had no significant difference. B, An in vivo imaging showed the average fluorescence area and intensity between vector group and PRRX1 overexpressed group intravenously via the tail vein after 4 wk. The fluorescence was mainly displayed in the lung tissue of PRRX1 overexpressed group. C, The body weight of the vector and PRRX1 overexpressed group intravenously via the tail vein after 4 wk. The average body weight was (19.90 ± 1.40) g in vector group, whereas (17.67 ± 1.20) g in PRRX1 overexpressed group. There was significantly different between these two groups (*P < .05). D, The haematoxylin and eosin staining of the lung and liver tissues in the vector and PRRX1 overexpressed group of nude mice intravenously via the tail vein after 4 wk (left ×200; right ×400). The top panel: the lung tissue of the vector group has no cancer cell; the middle panel: the liver tissue of the PRRX1 overexpressed group has no cancer cell; and the bottom panel: the lung tissue of the PRRX1 overexpressed group has cancer cells. E, The haematoxylin and eosin staining and immunohistochemistry of lung tissue in the PRRX1 overexpressed group of nude mice intravenously via the tail vein after 4 wk (×400). There was strong expression of PRRX1, while the weak expression of PPARG2 in PRRX1 overexpressed group (*P < .05). F, The GC/MS chromatograms of FFAs from primary and metastatic tumours with PRRX1 overexpressed in nude mice xenograft tumour model. Compared with the primary tumour, the concentration and category of FFAs in the metastatic tumour were significantly increased. The percentage of C14 reduced from 52.35% to 1.32%, while the percentage of C16 and C18 increased from 47.65% to 98.68% (**P < .01). Error bars represent the mean ± SD of triplicate experiments. Unpaired *t* test was performed to determine significance

We next examined whether the PRRX1/PPARG2 pathway observed in cell culture could modulate the metastasis of salivary adenoid cystic carcinoma in animal models. As shown in Figure 6A, there was no significant difference of tumour volume between PRRX1 overexpressed group and the vector in the xenograft nude mice model when 4 weeks after subcutaneously injecting SACC‐83 cells (*P* = .91, Figure 6A). This suggests that PRRX1 has no obvious effect of the growth of the primary SACC. The nude mice implanted with low metastasis cell line SACC‐83 via the tail vein did not produce spontaneous lung metastases (Figure 6A); however, 100% of the mice implanted with PRRX1 overexpressed SACC‐83 via the tail vein produced lung metastases under living imaging (Figures 6B,C). And HE staining confirmed that there were tumour metastatic lumps in the lung tissue of PRRX1 overexpressed group, while cancer cells were not found in the liver tissues. And there was no cancer metastatic cell in the lung and liver of the group of SACC‐83 via the tail vein (Figure 6D). These data suggested that PRRX1 contributed to the metastatic phenotype of SACC.

Further, we examined the expression of PRRX1 and PPARG2 in the metastatic lung tissue. The data showed that there was the strong expression of PRRX1, while the weak expression of PPARG2 (Figure 6E). Compared with the primary tumour in the group of injecting subcutaneously PRRX1 overexpressed SACC‐83 cells, the concentration and category of FFAs in the metastatic tumour with PRRX1 overexpressed SACC‐83 cells via the tail vein were significantly increased. The percentage of C14 reduced from 52.35% to 1.32%, while the percentage of C16 and C18 increased from 47.65% to 98.68% (Figure 6F, Table S8). Importantly, the ability of SACC‐83 cells with PRRX1‐overexpressed to metastasize to the lung in nude mice was significantly repressed by upregulation of PPARG2 with administration of Rosiglitazone. These data suggested that PRRX1 and PPARG2 were responsible for the metastasis formation of SACC in mice.

## 4 DISCUSSION

**Figure 7:**
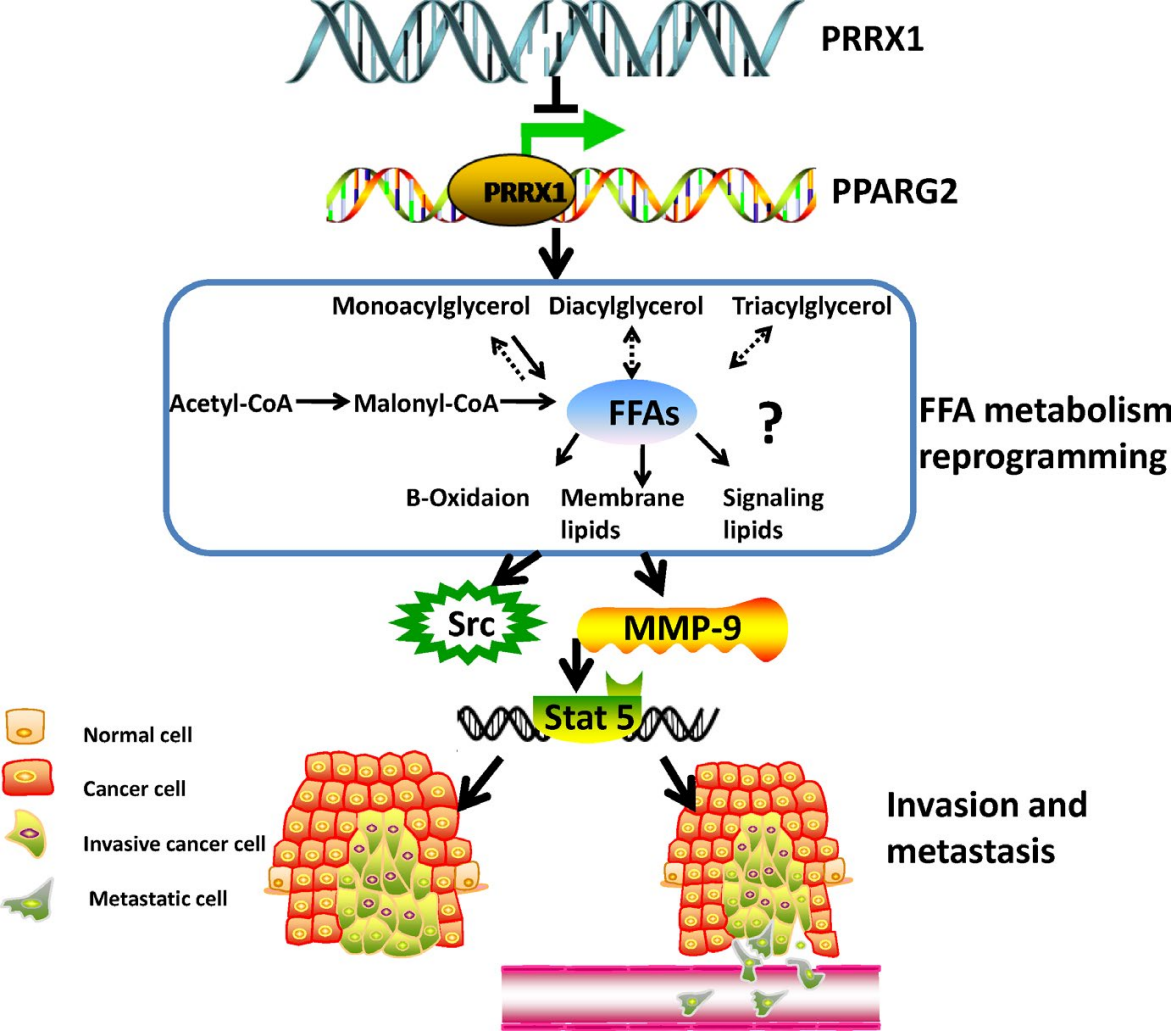
Schematic figure of PRRX1/PPARG2 pathway facilitated the invasion and metastasis of cancer cells. This schematic figure illustrated that PRRX1/PPARG2 pathway in cancer cells activated the metabolic reprogramming of FFAs to promote metastasis of SACC by Stat5‐DNA binding activity via Src and MMP‐9‐dependent pathway

Over the past decade, much research into carcinoma metastasis has focused on cancer cells themselves or their association with tumour immunity and inflammation microenvironment, without taking into much account the unique but complex tumour metabolic microenvironment.^32^ Evidence demonstrates a close correlation between EMT induction and lipid metabolic reprogramming in human cancer, which supports the energy requirements of increased motility and growth in harsh environmental condition.^33^ Our present study demonstrated that PRRX1 could contribute to the induction of EMT and the invasion and metastasis of cancer cells, recruiting FFAs accumulation in SACC. In SACC cells, the composition of FFAs in PRRX1‐overexpressed group was more complex, containing different kinds of FFAs and carbon chain extension. These changes were accompanied with an increase of the level of FFAs and a reduction of TG. Then, we found that overexpressed PRRX1 reduced the expression of PPARG2 in SACC cells and FFAs were induced in a PPARG2‐dependent manner. In turn, FFAs augment induced an increase of Stat5‐DNA binding activity via Src and MMP‐9. Moreover, high PRRX1 expression was significantly associated with high FFAs level and poor survival of SACC patients. We therefore suggested that PRRX1‐induced EMT in cancer cells activated the metabolic reprogramming of FFAs to promote metastasis of SACC (Figure 7). This is the first study, to our knowledge, suggesting EMT might be also thought to have another function: to create an aberrant metabolic microenvironment by educating FFAs, just like building an immunosuppressive microenvironment by recruiting the infiltrating inflammatory factors and immune cells.^34^

Recently, there has been a growing body of evidences showing that FFAs participated in tumour progression.^35^, ^36^ However, the precise role and molecular mechanism of metabolic reprogramming of FFAs during EMT and cancer metastasis remain unclear. In breast cancer cells, FASN was essential to induce EMT possibly through regulating L‐FABP, VEGF and VEGFR‐2.^24^ FASN knockdown with reducing FA accumulation cut down cancer cell proliferation and EMT execution, induced apoptosis, and decreased the sizes of colorectal, ovarian and breast cancer xenografts.^37^, ^38^, ^39^ Here, we showed that the FFAs levels of PRRX1‐silenced SACC cells decreased, accompanying with the downregulation of Snail1 and Vimentin, the increasing of E‐cadherin and the reduction of the migration and invasion of cancer cells, while FFAs of PRRX1 overexpressed SACC cells induced EMT and promoted the migration and invasion of cancer cells. This indicated that EMT programme activated the alteration of FFAs to promote invasion and migration of SACC cells. In addition, the study of the metastatic MDA‐MB‐231 breast cancer cells showed that hPEBP4 promoted the expression or activity of MMP2, MMP 9 and MMP13 through an increase association of Akt with Src.^40^ Stimulation of MDA‐MB‐231 with oleic acid induces an increase of MMP9 secretion through a PKC, Src, and EGFR‐dependent pathway.^31^ Here, we found that tumour‐derived FFAs activated Src and MMP‐9 to induce an increase of Stat5‐DNA to enable tumour migration and invasion, suggesting that FFAs are the novel and clinically relevant metastatic accelerator of SACC. Our results, along with previous reports, suggest that MMP9 and Src are involved in tumour FFAs metabolic microenvironment and are important for FFAs to contribute to tumour spread. All above indicated that EMT in cancer cells might be causative, not just a bystander in aberrant metabolic microenvironment by educating FFAs.

The family of paired‐related homeobox transcription factors includes PRRX1a, PRRX1b and PRRX2. PRRX1 has been shown to be closely related to EMT process and the tumour invasion and metastasis.^19^, ^20^ In colorectal cancer cases, PRRX1 is an indicator of metastasis and poor prognosis.^41^ In gastric carcinoma, the high expression of PRRX1 promoted the EMT process through mediating Wnt/β‐catenin signalling.^42^ In contrast, in hepatocellular carcinoma, downregulation of PRRX1 expression contributes to the poor prognosis of patients through acquisition of CSC‐like properties.^43^ PRRX1 is an EMT inducer conferring migratory and invasive properties in the primary tumour, and the loss of PRRX1 is required for cancer cells to metastasize in metastatic colonization in vivo, since the loss of PRRX1 could revert to the epithelial phenotype concomitant with the acquisition of stem cell properties.^44^ In this article, we showed that PRRX1 induced EMT and added the migration and invasion of SACC cells and promoted the clone and metastatic ability of lung metastases in vivo. And PRRX1 was positively associated with the poor prognosis in SACC patients. Considering the variations among different types of tumours, there may be tissue‐specific mechanisms of PRRX1 regulation in response to cancer metastasis and prognosis.

On the other side, PRRX1 is implicated with regulation of mesenchymal cell fate, including myogenesis and skeletogenesis and adipogenesis.^45^ PRRX1 could influence PPARG, CCAAT/enhancer‐binding protein‐α, FABP4, adiponectin and chemerin.^46^ However, whether PRRX1 impacts tumour‐induced FFAs remains to be addressed. This is the first study, to our knowledge, to investigate the effect of PRRX1 on tumour‐induced FFAs and we found that overexpressed PRRX1 increased FFAs levels and promoted the invasion and migration of SACC cells. Conversely, silencing PRRX1 reduced the FFAs levels and inhibited the invasion and migration of SACC cells. This indicated that PRRX1 activated the metabolism of FFAs to promote the invasion and migration of SACC cells.

Peroxisome proliferator–activated receptor (PPAR) has PPAR‐α, PPAR‐β and PPAR‐γ, and PPARG2 is a subtype of PPAR‐γ. Previous studies have demonstrated that PPARG2, high expression in large intestine, adipose tissue and hematopoietic cells, is known to activate adipocyte genes.^29^ Recently, PPARγ expression has been described in a variety of human malignancies, including breast cancer, prostate cancer, glioblastoma, non–small cell lung carcinoma, ovarian cancer and pancreatic carcinoma.^30^ However, the current knowledge about the correlation between PPARG2 and metabolic syndromes in SACC is still missing. We found that in SACC cells, PPARG2 agonist led to the lower concentration of FFAs, and the lower migration and invasion abilities. In contrast, PPARG2 antagonist generated higher FFA levels, and the higher cell migration and invasion abilities. These suggested that FFAs promoted the invasion and migration of SACC cells in a PPARG2‐dependent manner. These results are supported by the previous study of Forootan et al,^47^ who showed that the excessive amount of fatty acids transported by FABP5 may facilitate the malignant progression of prostate cancer cells through FABP5‐PPARγ‐VEGF signalling. In addition, PPARG2 risk loci analysis showed that PRRX1 can be combined with rs4684847C to inhibit the expression of PPARG2.^20^ UniHI protein interaction database predicted the existence of a correlation between PRRX1 and PPARG2.^48^ Here, our experiments showed that overexpression of PRRX1 inhibited the expression of PPARG2 and knockdown of PRRX1 increased the expression of PPARG2 in SACC cells. The results of ChIP further confirmed PRRX1‐pparg2 compound significantly increased in SACC‐83 and SACC‐LM cells with PRRX1 overexpression. Our results, along with previous reports, indicated that PRRX1 can directly regulate the expression of PPARG2 in FFAs accumulation to contribute to the migration and invasion of SACC.

Our results provide new insights into the action of PRRX1 as a link of EMT and FFAs metabolism in salivary adenoid cystic carcinoma. The PRRX1‐induced EMT of cancer cells helps to educate newly recruited FFAs by targeting PPARG2. FFAs create metabolism conditions in the tumour microenvironment and promote metastasis. Preventing PRRX1 elevation may stop the creation of a disorder metabolism microenvironment and slow metastasis. Therefore, targeting PRRX1 may be a promising strategy for treating human salivary adenoid cystic carcinoma in the future.

## CONFLICT OF INTEREST

The authors declare no conflicts of interest.

## Data Availability

The data that support the findings of this study are available from the corresponding author upon reasonable request.
