# Supplementary figures and images for: PRRX1‐induced epithelial‐to‐mesenchymal transition in salivary adenoid cystic carcinoma activates the metabolic reprogramming of free fatty acids to promote invasion and metastasis
Source: Cell Prolif. 2019 Oct 27;53(1):e12705. doi: 10.1111/cpr.12705 (PMC6985691; doi:10.1111/cpr.12705)

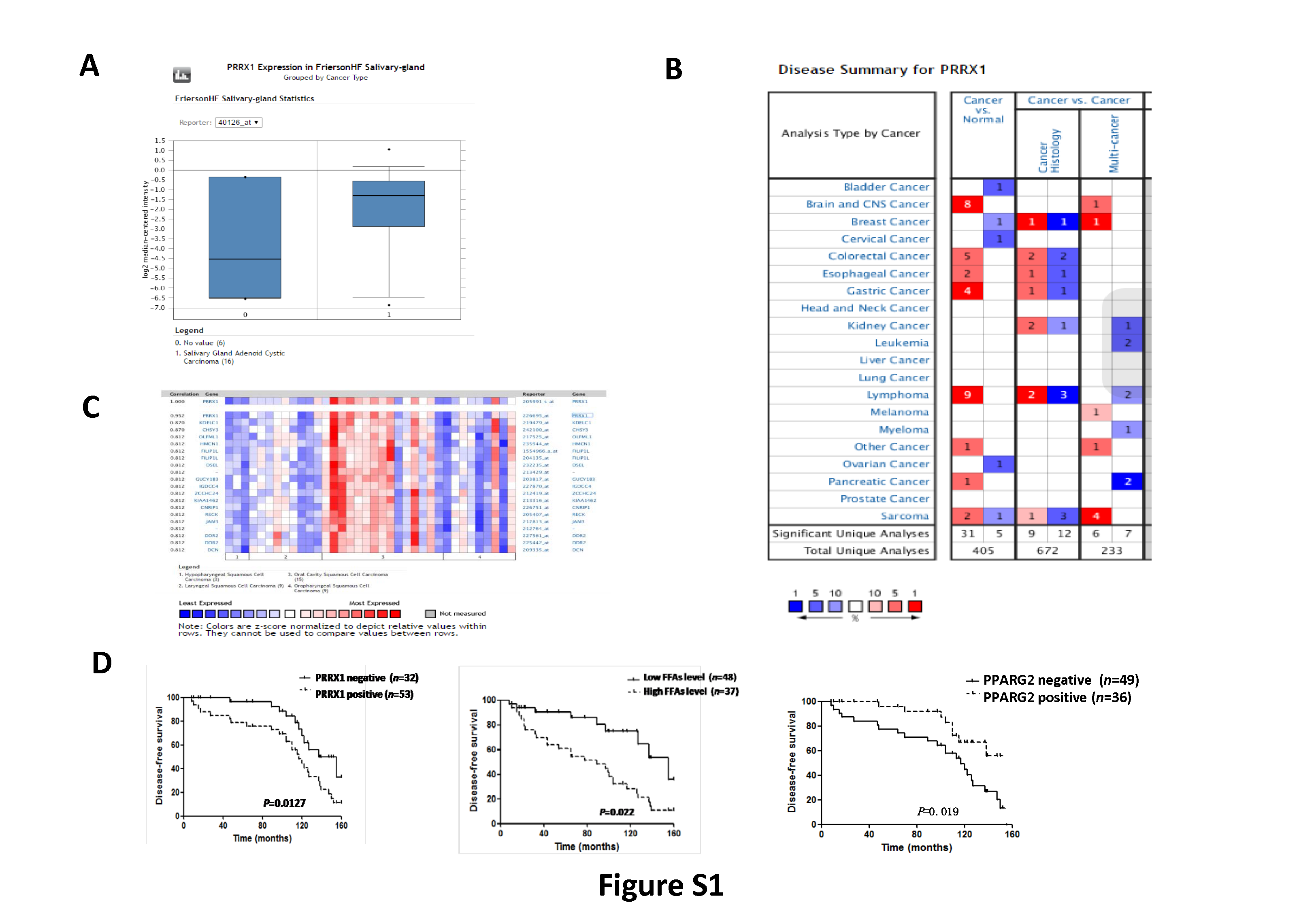

Supplement: Supplementary file 1 [file CPR-53-e12705-s001.tiff]

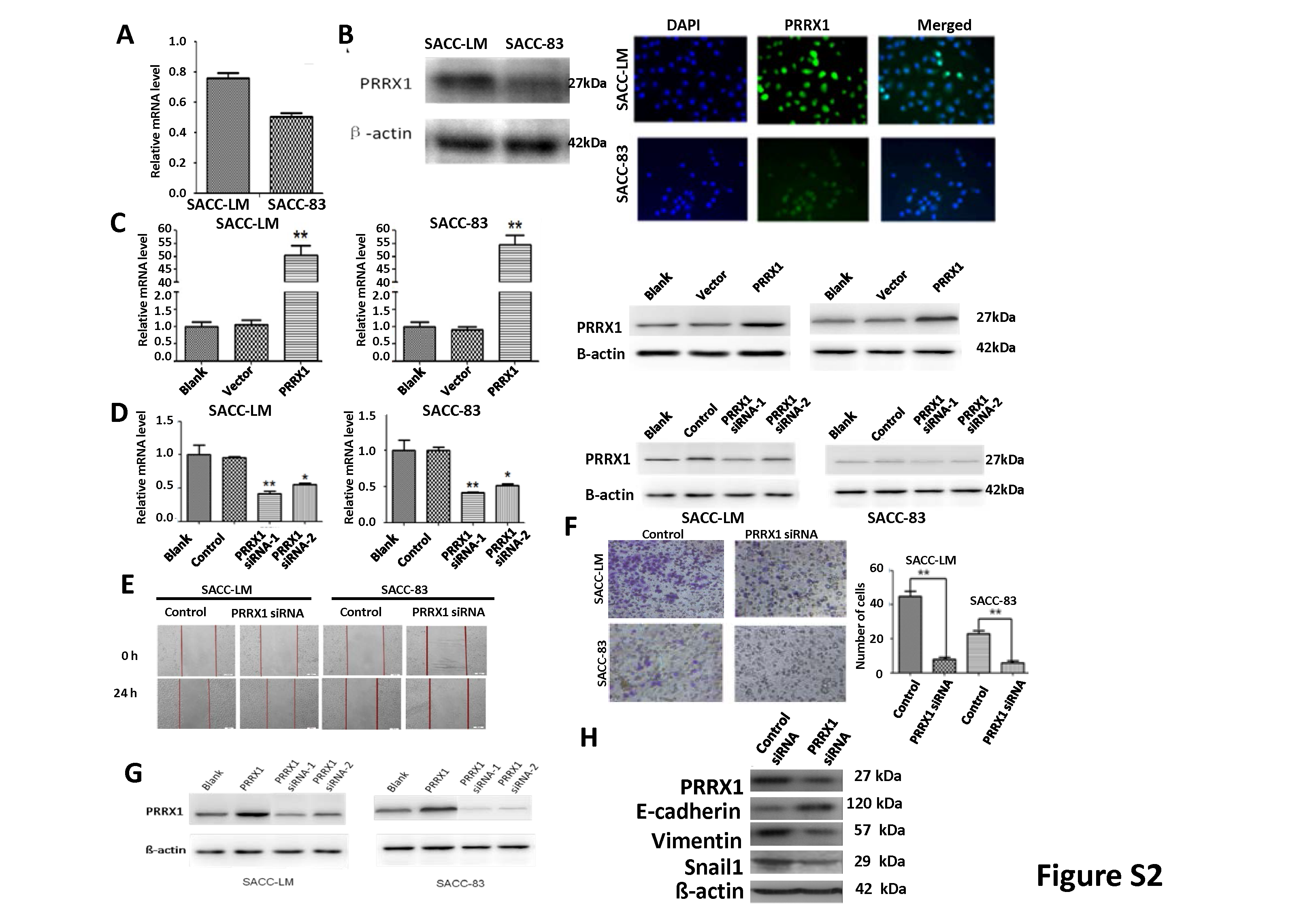

Supplement: Supplementary file 2 [file CPR-53-e12705-s002.tiff]

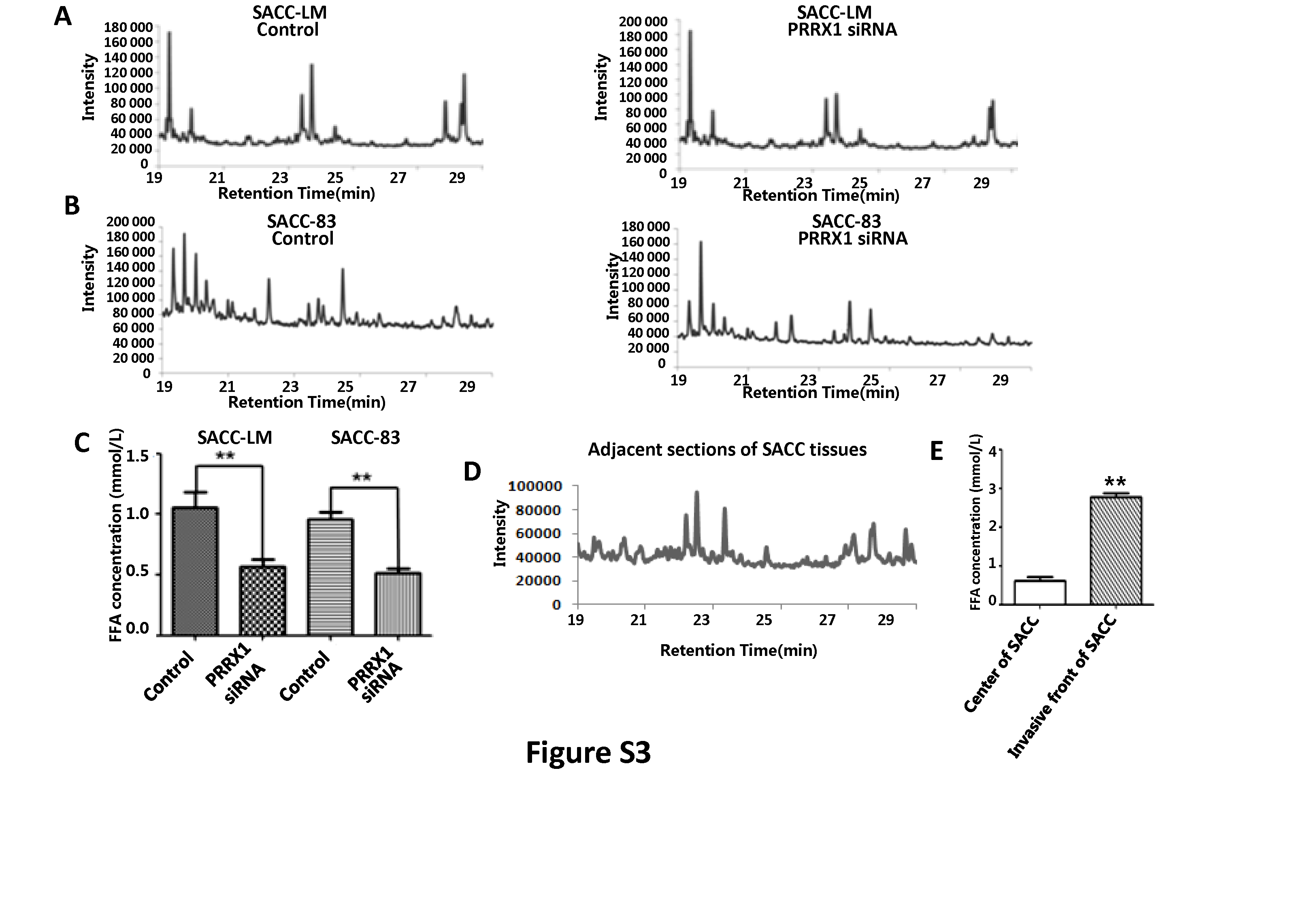

Supplement: Supplementary file 3 [file CPR-53-e12705-s003.tiff]

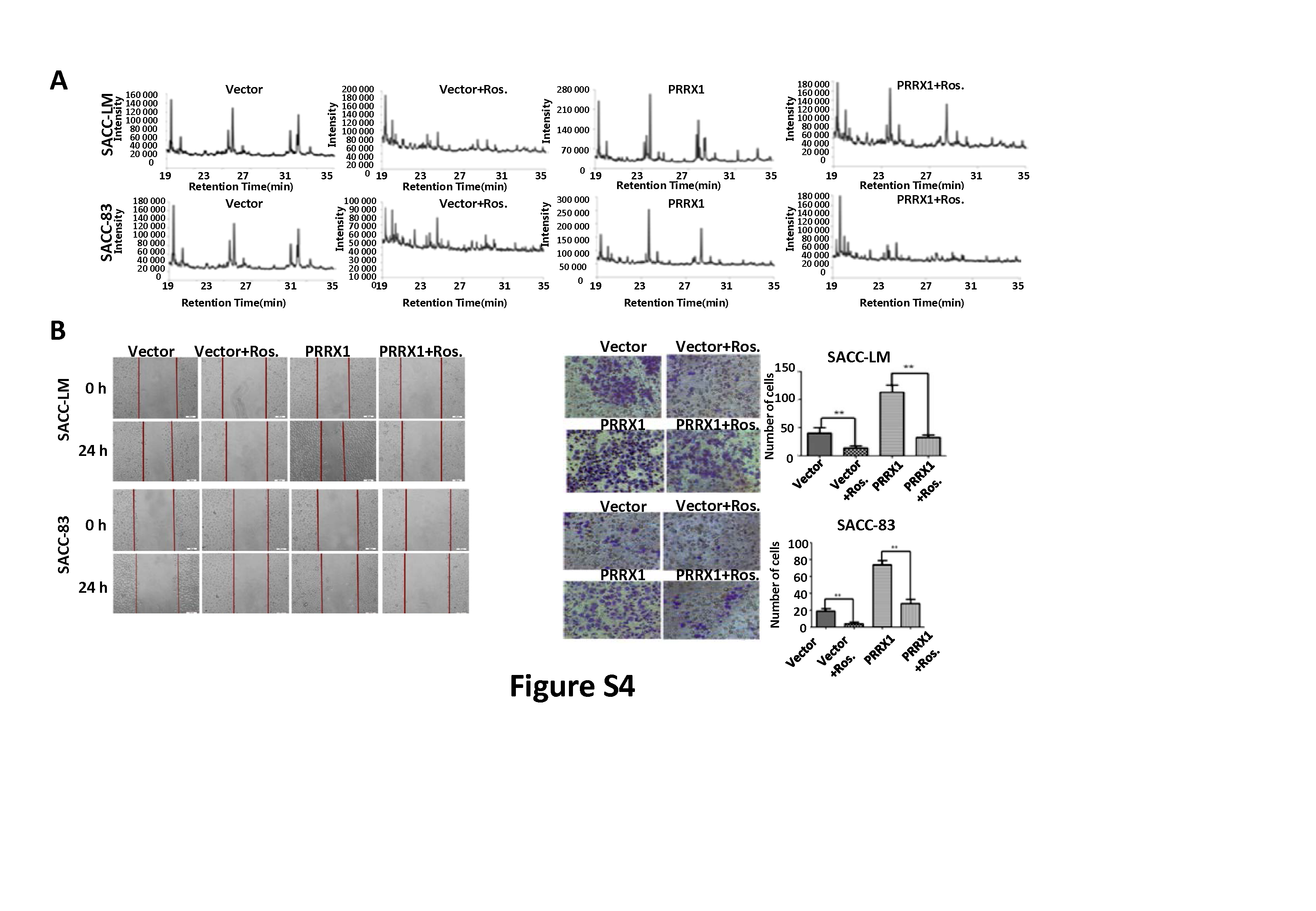

Supplement: Supplementary file 4 [file CPR-53-e12705-s004.tiff]

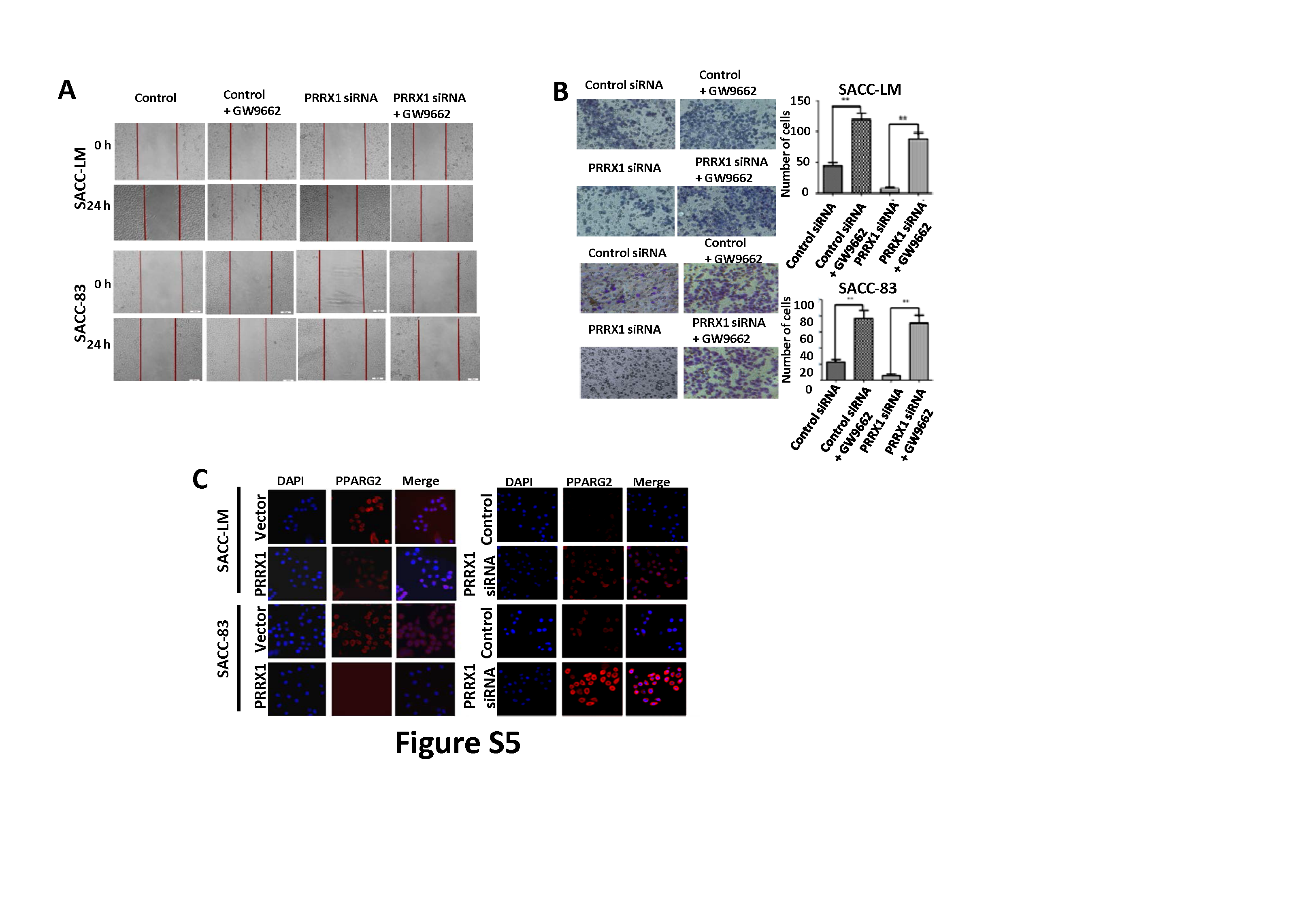

Supplement: Supplementary file 5 [file CPR-53-e12705-s005.tiff]

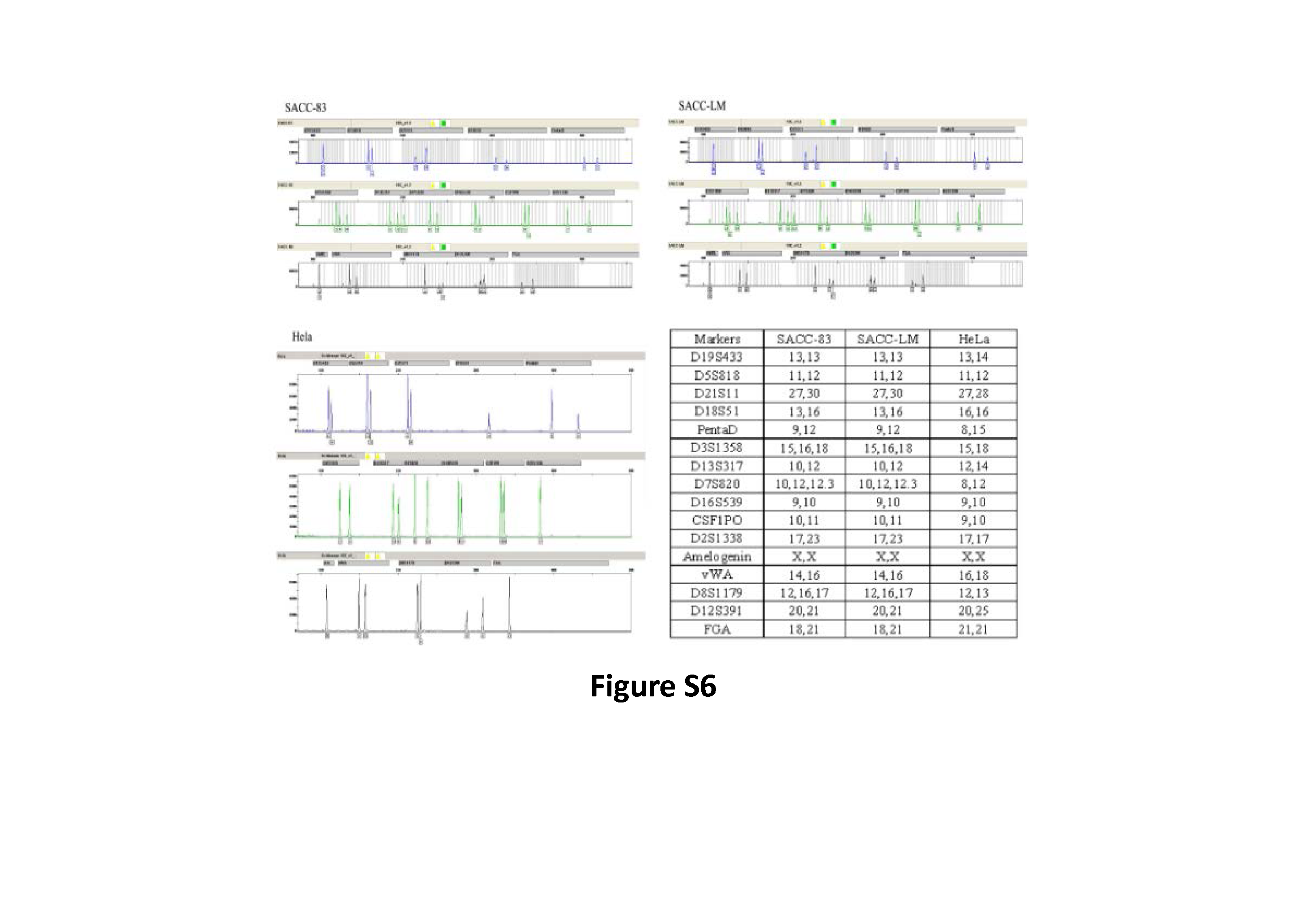

Supplement: Supplementary file 6 [file CPR-53-e12705-s006.tiff]
