# Supplementary material for: PRRX1‐induced epithelial‐to‐mesenchymal transition in salivary adenoid cystic carcinoma activates the metabolic reprogramming of free fatty acids to promote invasion and metastasis
Source: Cell Prolif. 2019 Oct 27;53(1):e12705. doi: 10.1111/cpr.12705 (PMC6985691; doi:10.1111/cpr.12705)
Supplement: Supplementary file 7 [file CPR-53-e12705-s007.docx]

**Table 1. Clinical-pathologic characteristic of 85 patients with SACC, and association between PRRX1expression and these variables**

| **Clinical-pathologic variables** | **NO. of patients** | **PRRX1 expression** | | | | | | ***P* value** | |  |
| --- | --- | --- | --- | --- | --- | --- | --- | --- | --- | --- |
|  |  | **Negative (%)** | | **Positive (%)** | | | |  |  |  |
| **Age** | 0.819 | | | | | | | | |  |
| ＜50 | 34 | | 13(38.24) | 21(61.76) | | |  | | |  |
| ≥50 | 51 | | 19(37.25) | 32(62.75) | | |  | | |  |
| **Gender** | 0.824 | | | | | | | | |  |
| male | 47 | | 17(36.17) | 30(63.83) | | |  | | |  |
| female | 38 | | 15(39.47) | 23(60.53) | | |  | | |  |
| **Location** |  | |  |  | | | **0.013** | | |  |
| major | 40 | | 21(52.50) | 19(47.50) | | |  | | |  |
| minor | 45 | | 11(24.44) | 34(75.56) | | |  | | |  |
| **Histological subtype** | **0.024** | | | | | | | | |  |
| pore/tube | 62 | | 28(45.16) | 34(54.84) | | |  | | |  |
| solid | 23 | | 4(17.39) | 19(82.61) | | |  | | |  |
| **TNM stage** |  | |  |  |  |  | | | **0.015** | |
| I-II | 26 | | 15(57.69) | 11(42.31) | | |  | | |  |
| III-IV | 59 | | 17(28.81) | 42(71.19) | | |  | | |  |
| **Perineural invasion** |  | |  |  |  |  | | | **0.014** | |
| positive | 38 | | 18(47.37) | 20(52.63) | | |  | | |  |
| negative | 47 | | 35(74.47) | 12(25.53) | | |  | | |  |
| **Metastasis** |  | |  |  |  |  | | | **0.003** | |
| positive | 33 | | 14(42.42) | 19(57.58) | | |  | | |  |
| negative | 52 | | 39(75.00) | 13(25.00) | | |  | | |  |
| **Recurrence** |  | |  |  |  |  | | | **0.043** | |
| positive | 23 | | 10(43.48) | 13(56.52) | | |  | | |  |
| negative | 62 | | 43(69.35) | 19(30.65) | | |  | | |  |

**Table S1 The FFAs composition of SACC patients and normal group**

| **FFAs composition** | **Normal group (%)** | **SACC patients (%)** | |
| --- | --- | --- | --- |
|  |  | **PRRX1 negative** | **PRRX1 positive** |
| C14：0 | 88.20±5.52 | 0 | 0 |
| C16：0 | 1.80±1.03 | 93.55±6.58 | 52.94±3.30 |
| C18：0 | 0 | 6.45±0.58 | 20.59±1.79 |
| C18：2 | 0 | 0 | 9.32±0.89 |
| C18：1 | 0 | 0 | 17.15±1.66 |

**Table S2 The FFAs composition of the PRRX1 overexpressed SACC cells and the control**

| **FFA****s composition** | **SACC-LM** | | **SACC-83** | |
| --- | --- | --- | --- | --- |
|  | **Vector（%）** | **PRRX1-OVER (%)** | **Vector（%）** | **PRRX1-OVER (%)** |
| C14：0 | 0 | 1.28±0.12 | 82.56±5.51 | 0.79±0.04 |
| C16：0 | 40.64±3.32 | 41.37±2.94 | 17.44±1.02 | 50.52±3.95 |
| C16：1 | 0 | 8.80±0.63 | 0 | 0 |
| C17：0 | 0 | 11.63±0.93 | 0 | 0 |
| C18：0 | 35.34±2.25 | 0 | 0 | 34.02±1.92 |
| C18：1 | 24.02±1.41 | 24.87±1.35 | 0 | 9.86±0.87 |
| C18：2 | 0 | 12.04±1.21 | 0 | 4.81±0.53 |

**Table S3 The FFAs composition of the PRRX1 silenced SACC cells and the control**

| **FFAs composition** | **SACC-LM** | | **SACC-83** | |
| --- | --- | --- | --- | --- |
|  | **Control siRNA(%)** | **siRNA-1 (%)** | **Control siRNA(%)** | **siRNA-1(%)** |
| C14：0 | 0 | 0 | 71.63±4.18 | 91.38±5.52 |
| C16：0 | 41.50±3.27 | 52.27±4.31 | 28.37±1.29 | 8.62±0.87 |
| C18：1 | 23.63±1.47 | 7.41±0.67 | 0 | 0 |
| C18：0 | 34.87±1.94 | 40.32±3.54 | 0 | 0 |

**Table S4 The FFA composition of the Rosiglitazone stimulation in SACC-LM cells**

| **FFAs composition** | **Vector（%）** | **Vector**  **+** [**rosiglitazone**](javascript:void(0);)  **(%)** | **PRRX1-OVER（%）** | **PRRX1-OVER**  **+**[**rosiglitazone**](javascript:void(0);) **(%)** |
| --- | --- | --- | --- | --- |
| C14:0 | 0 | 0 | 1.28±0.12 | 0.83±0.07 |
| C16:0 | 40.64±3.32 | 100 | 41.37±2.94 | 57.85±4.64 |
| C16:1 | 0 | 0 | 8.80±0.63 | 0 |
| C17:0 | 0 | 0 | 11.63±0.93 | 0 |
| C18:0 | 35.34±2.25 | 0 | 0 | 41.32±2.89 |
| C18:1 | 24.02±1.41 | 0 | 24.87±1.35 | 0 |
| C18:2 |  | 0 | 12.04±1.21 | 0 |

**Table S5 The FFAs composition of the Rosiglitazone stimulation in SACC-83 cells**

| **FFA composition** | **Vector（%）** | **Vector**  **+** [**rosiglitazone**](javascript:void(0);)  **(%)** | **PRRX1-OVER（%）** | **PRRX1-OVER**  **+**[**rosiglitazone**](javascript:void(0);) **(%)** |
| --- | --- | --- | --- | --- |
| C14:0 | 82.56±5.51 | 0 | 0.79±0.04 | 69.34±4.54 |
| C15:0 | 0 | 0 | 0 | 0 |
| C16:0 | 17.44±1.02 | 100 | 50.52±3.95 | 21.37±1.81 |
| C18:0 | 0 | 0 | 34.02±1.92 | 9.29±0.89 |
| C18:1 | 0 | 0 | 9.86±0.87 | 0 |
| C18:2 | 0 | 0 | 4.81±0.53 | 0 |

**Table S6 The FFAs composition of the GW9662 stimulation in SACC-LM cells**

| **FFAs composition** | **Control siRNA**  **(%)** | **Control siRNA**  **+ GW 9662(%)** | **PRRX1siRNA-1 (%)** | **PRRX1siRNA-1**  **+ GW 9662 (%)** |
| --- | --- | --- | --- | --- |
| C14:0 | 0 | 1.23±0.16 | 0 | 1.27±0.11 |
| C15:0 | 0 | 0.49±0.07 | 0 | 0.31±0.02 |
| C16:0 | 41.50±3.27 | 42.25±4.16 | 52.27±4.31 | 40.46±2.75 |
| C16:1 | 0 | 0 | 0 | 0 |
| C17:0 | 0 | 0 | 0 | 0 |
| C18:0 | 34.87±1.94 | 13.85±1.28 | 40.32±3.54 | 16.95±0.13 |
| C18:1 | 23.63±1.47 | 30.74±2.94 | 7.41±0.67 | 33.22±0.20 |
| C18:2 | 0 | 2.09±0.19 | 0 | 1.61±0.14 |
| C19:1 | 0 | 6.70±0.48 | 0 | 6.18±0.54 |
| C20:1 | 0 | 0.44±0.03 | 0 | 0 |
| C20:4 | 0 | 2.22±0.13 | 0 | 0 |

**Table S7 The FFAs composition of the GW9662 stimulation in SACC-83 cells**

| **FFAs composition** | **Control siRNA**  **(%)** | **Control siRNA**  **+ GW 9662(%)** | **PRRX1siRNA-1 (%)** | **PRRX1siRNA-1**  **+ GW 9662 (%)** |
| --- | --- | --- | --- | --- |
| 14:0 | 71.63±5.18 | 0.96±0.08 | 91.38±5.52 | 0.3±0.02 |
| C15:0 | 0 | 0.54±0.04 | 0 | 0 |
| C16:0 | 28.37±1.29 | 35.20±2.27 | 8.62±0.87 | 33.31±2.45 |
| C18:0 | 0 | 17.68±1.63 | 0 | 30.78±1.99 |
| C18:1 | 0 | 30.22±2.69 | 0 | 18.47±1.68 |
| C18:2 | 0 | 4.91±0.04 | 0 | 4.85±0.03 |
| C19:1 | 0 | 3.4 ±0.03 | 0 | 0 |
| C20:0 | 0 | 0.25±0.01 | 0 | 0 |
| C20:1 | 0 | 6.84±0.51 | 0 | 12.28±0.11 |

**Table S8 The FFAs composition of in the metastatic tumor of nude mice xenograft with PRRX1 overexpressed SACC-83 via the tail vein**

| **FFAs composition** | **Vector（%）** | **PRRX1-Overexpression(%)** |
| --- | --- | --- |
| C14:0 | 52.35±4.72 | 1.32±0.10 |
| C16:0 | 26.88±1.32 | 44.50±3.23 |
| C16:1 | 0 | 7.32±0.54 |
| C18:0 | 0 | 11.43±1.01 |
| C18:1 | 20.78±1.30 | 23.66±1.22 |
| C18:2 | 0 | 11.78±1.09 |
